# Mapping the *O*-Mannose Glycoproteome in *Saccharomyces cerevisiae* * [Fn FN2]

**DOI:** 10.1074/mcp.M115.057505

**Published:** 2016-01-13

**Authors:** Patrick Neubert, Adnan Halim, Martin Zauser, Andreas Essig, Hiren J. Joshi, Ewa Zatorska, Ida Signe Bohse Larsen, Martin Loibl, Joan Castells-Ballester, Markus Aebi, Henrik Clausen, Sabine Strahl

**Affiliations:** From the ‡Centre for Organismal Studies (COS), Department of Cell Chemistry, Heidelberg University, Im Neuenheimer Feld 360, D-69120 Heidelberg, Germany;; §Copenhagen Center for Glycomics, Departments of Cellular and Molecular Medicine, Faculty of Health Sciences, University of Copenhagen, Blegdamsvej 3, DK-2200 Copenhagen N, Denmark;; ¶Institute of Microbiology, Department of Biology, Swiss Federal Institute of Technology, ETH Zürich, CH-8093 Zürich, Switzerland

## Abstract

*O*-Mannosylation is a vital protein modification conserved from fungi to humans. Yeast is a perfect model to study this post-translational modification, because in contrast to mammals *O*-mannosylation is the only type of *O*-glycosylation. In an essential step toward the full understanding of protein *O*-mannosylation we mapped the *O*-mannose glycoproteome in baker's yeast. Taking advantage of an *O*-glycan elongation deficient yeast strain to simplify sample complexity, we identified over 500 *O*-glycoproteins from all subcellular compartments for which over 2300 *O*-mannosylation sites were mapped by electron-transfer dissociation (ETD)-based MS/MS. In this study, we focus on the 293 *O*-glycoproteins (over 1900 glycosylation sites identified by ETD-MS/MS) that enter the secretory pathway and are targets of ER-localized protein *O*-mannosyltransferases. We find that *O*-mannosylation is not only a prominent modification of cell wall and plasma membrane proteins, but also of a large number of proteins from the secretory pathway with crucial functions in protein glycosylation, folding, quality control, and trafficking. The analysis of glycosylation sites revealed that *O*-mannosylation is favored in unstructured regions and β-strands. Furthermore, *O*-mannosylation is impeded in the proximity of *N*-glycosylation sites suggesting the interplay of these types of post-translational modifications. The detailed knowledge of the target proteins and their *O*-mannosylation sites opens for discovery of new roles of this essential modification in eukaryotes, and for a first glance on the evolution of different types of *O*-glycosylation from yeast to mammals.

Protein glycosylation is one of the most diverse, complex, and energetically costly post-translational modifications. The most common types are *N*- and *O*-glycosylation with glycans attached to the amide group of Asn residues of the sequon Asn-X-Ser/Thr, and to the hydroxy amino acids Ser and Thr, respectively. Although various types of *O*-glycosylation have evolved in mammals (*i.e. O*-linked *N*-acetylgalactosamine (*O*-GalNAc), *N*-acetylglucosamine, xylose, fucose, or glucose), only *O*-mannosylation is conserved between fungi, animals, and humans (1). In higher eukaryotes, *O*-mannosylation is essential for growth and development, and failures result in severe congenital human disorders, stressing its importance (2–5). In baker's yeast, *O*-mannosylation is equally essential and it is the only identified type of protein *O*-glycosylation making *Saccharomyces cerevisiae* an ideal model to study this post-translational modification. However, still very little is known about the *O*-mannose (*O*-Man)^1^ glycoproteome as well as the biological functions of *O*-mannosyl (*O*-Man) glycans.

In eukaryotes, biosynthesis of *O*-Man glycans on proteins trafficking the secretory pathway is initiated at the luminal side of the endoplasmic reticulum (ER) membrane by the conserved family of protein *O*-mannosyltransferases (PMTs, in mammals called POMTs) that catalyze the transfer of mannose from dolichol-phosphate β-d-mannose, an activated lipid-linked mannosyl donor, to the substrate glycoproteins (6, 7). This reaction takes place at the ER translocon on co- and post-translationally translocating nascent polypeptides, as we recently showed in baker's yeast (8). While traveling through the secretory pathway from the ER to later destinations, the protein-linked mannose residues can be further extended by the addition of different sugar residues, giving rise to more complex glycan structures that vary between species. In baker's yeast, *O*-Man glycan elongation is mediated by the α1,2-mannosyltransferases of the KTR family (*i.e.* Kre2, Ktr1, Ktr3) and the α1,3-mannosyltansferases from the MNN1 family in the Golgi apparatus (9). As a result linear oligo-mannose structures of heterogenous length arise (10).

To our best knowledge *O*-mannosylation of less than 40 yeast proteins, mainly including extracellular, cell wall-localized, and plasma membrane proteins have been shown so far (reviewed in (11)). In addition, only about 90 *O*-Man glycosites have been experimentally assigned, all in only seven cell wall proteins (Aga1, Ccw5, Ccw12–14, Sag1, Sed1; (12–16)). Most of these *O*-Man glycans are situated in Ser/Thr-rich protein regions. Although around 18% of signal peptide-containing proteins have been predicted by computational analyses to contain at least one Ser/Thr-rich region, exact motifs that determine PMT-based *O*-mannosylation have only been explored in part (6, 17–19).

Proteome-wide analysis of *O*-glycosylation has long been limited by technical constraints but recent advances in enrichment strategies of glycopeptides followed by MS sequencing have paved the way for *O*-glycoproteomics studies (20). We recently characterized the human *O*-Man glycoproteome taking advantage of the so-called “SimpleCell” strategy that resulted in the identification of cadherins as major targets of *O*-mannosylation in mammals (21). Key to this study was the use of a genetically engineered cell line where early glycan elongation steps in the Golgi apparatus are omitted to simplify and hence effectively reduce the sample complexity. The “SimpleCell” strategy enabled sensitive isolation and sequencing of *O*-glycopeptides using a combination of lectin weak affinity chromatography (LWAC) and higher-energy collision-induced dissociation-electron-transfer dissociation (HCD/ETD)-based MS/MS (20, 21).

Here, we took advantage of a yeast mutant in which *O*-linked mannose residues are barely extended in the Golgi apparatus to determine the first comprehensive *O*-Man glycoproteome in *Saccharomyces cerevisiae*. We analyzed total cell lysates using the shotgun LWAC enrichment strategy developed for the human *O*-Man glycoproteome to obtain a global view. In addition, we applied a targeted approach to probe cell wall proteins specifically, which validated the sensitivity and robustness of the shotgun strategy. This way, we identified 511 *O*-Man glycoproteins from all major cellular compartments including a subset of nucleocytoplasmic proteins that is discussed in a separate study (22). Here, we focus on 293 proteins that enter the secretory pathway and thus are glycosylated by the ER-located PMTs. For these proteins, we identified over 1900 *O*-Man sites by ETD-MS/MS and over 260 additional *O*-Man sites by HCD-MS/MS. The large dataset identified numerous previously unknown targets and revealed hallmarks of PMT-based protein *O*-mannosylation. Our study gives insight into conserved features of *O*-glycosylation between yeast and mammals.

## EXPERIMENTAL PROCEDURES

### 

#### 

##### Yeast Strains and Plasmids

*S. cerevisiae* strains and plasmids used in this study are summarized in supplemental Table S1 and Supplemental Experimental Procedures. Yeast cells were grown in YPD (1% yeast extract, 2% peptone, 2% glucose) under standard conditions.

##### Preparation of Shotgun LWAC-enriched O-Man Glycopeptides from Total Cell Lysates

Total cell lysates were prepared from a total of 100 OD_600_ units of mid-log phase yeast cells (strains BY4741 and *KTR*Δ) with 0.25–0.5 mm glass beads in 400 μl of ice-cold 0.1% (w/v) Rapigest (Waters Corp., Milford, MA) in 50 mm NH_4_HCO_3_ using a Hybaid RiboLyser (Thermo Fisher Scientific, Bonn, Germany; 4 × 25 s with 1 min intervals at 4 °C). After cell lysis, the bottom of the tube was punctured, and the lysate was collected. Cell debris was removed by centrifugation at 1500 × *g* for 5 min at 4 °C. Cleared lysates were heated at 80 °C for 10 min, followed by reduction in 5 mm DTT at 60 °C for 30 min and alkylation in 10 mm iodoacetamide at room temperature for 30 min. Samples were digested with either 25 μg trypsin (Roche Diagnostics, Mannheim, Germany), 20 μg GluC (*Staphylococcus aureus* Protease V8), or 25 μg chymotrypsin (Promega, Madison, WI) over night. After incubation at 95 °C for 20 min, samples were treated with 8 U Peptide-N-Glycosidase F (PNGase F; Roche Diagnostics) over night at 37 °C. An additional 4 U PNGase F was added and samples incubated for additional 4h at 37 °C. The digests were acidified with 12 μl TFA, incubated at 37 °C for 20 min, cleared by centrifugation at 10,000 × *g* for 10 min, and purified by Sep-Pak C18 (Waters, Dublin, Ireland) columns. Purified peptides were dried by evaporation, and then solved in 2 × Con A buffer A (21). *O*-Man glycopeptides were enriched by LWAC and further fractionated by isoelectric focusing (IEF) as previously described (21, 23). MS analysis was performed on Con A-enriched glycopeptide fractions prepared from wild-type (WT) and KTRΔ cells. In total, data were collected from eight independent preparations. Total cell lysates from *KTR*Δ were digested by trypsin in triplicates, and digested by GluC and chymotrypsin in duplicates each. Additionally, data were collected from a single WT cell extract digested by trypsin.

##### Preparation of (Glyco-)Peptides from Enriched Covalently Linked Cell Wall Proteins

The protocol was adapted from Schulz and Aebi (24) and optimized for ETD-MS/MS measurements of *O*-mannosylated cell wall proteins. Briefly, 50 OD_600_ units of cells (strain SS328) were used to prepare total cell lysates. Cell walls were collected and resuspended in denaturing buffer (50 mm Tris-HCl, pH 7.5, 2 m thiourea, 7 m urea, 2% (w/v) SDS). Cell wall proteins were reduced by DTT, and after alkylation the cell wall pellet was extensively washed with denaturing buffer and subsequently with 2% (w/v) SDS. The pellet was then dissolved in 2% SDS and the *N*-glycans trimmed down to a single HexNAc by the endoglycosidase H (Endo H) for 16 h according to the manufacturer's protocol (New England Biolabs, Frankfurt/Main, Germany). After washing and dissolving the pellet in 50 mm Na_4_HCO_3_ (pH 8), the cell wall proteins were digested with either AspN (2 μg/ml; Merck Millipore, Darmstadt, Germany), LysC (20 μg/ml; Wako, Osaka, Japan), or LysN (6 μg/ml; U-Protein Express BV, Utrecht, Netherlands) at 37 °C for 16 h. The released peptides were separated from the remaining cell wall material by centrifugation at 16,000 × *g* for 1 min, and completely dried in a centrifugal evaporator. The peptides were dissolved in 100 mm sodium acetate buffer (pH 4.6) and treated with α-mannosidase from Jack bean at 37 °C for 16 h. Afterward, the peptide solution was acidified with formic acid to pH 2–3 and desalted using C18 ZipTip pipette tips (Merck Millipore). For the LysN digest, HCD-MS/MS was performed with the same extract as prepared for ETD. For the LysC digest, the ETD and HCD measurements were performed with two independently prepared extracts. ETD was solely used for the extract digested with AspN.

##### nLC-MS/MS

MS analyses of IEF fractions of *O*-Man glycopeptides enriched from total cell lysates by Con A LWAC were performed essentially as previously described (21). Briefly, samples were analyzed on a set up composed of an EASY-nLC 1000 (Thermo Fisher Scientific, Bremen, Germany) interfaced via a nanoSpray Flex ion source to an LTQ-Orbitrap Velos Pro hybrid spectrometer (Thermo Fisher Scientific) or Fusion Tribrid mass spectrometer (Thermo Fisher Scientific). The EASY-nLC 1000 was operated using a single analytical column setup (PicoFrit Emitters, 75 μm inner diameter; New Objectives, Woburn, MA) packed in-house with Reprosil-Pure-AQ C18 phase (1.9 μm particle size; Dr. Maisch, Ammersbach, Germany). Peptides were separated using a 120 min LC gradient operated at 200 nL/min. The mobile phases were composed of solvent A (H_2_O) and solvent B (acetonitrile); both solvents containing 0.1% formic acid (v/v). The gradient was 2–25% B for 95 min followed by 25–80% B for 10 min and finally 80% B for 15 min. For Velos Pro acquisition, precursor MS1 scan (*m*/*z* 350–1700) was acquired in the Orbitrap at a resolution setting of 30,000, followed by Orbitrap HCD-MS/MS and ETD-MS/MS of the five most abundant multiply charged precursors in the MS1 spectrum; a minimum MS1 signal threshold of 50,000 ions was used for triggering data-dependent fragmentation events; MS2 spectra were acquired at a resolution of 15,000. For Fusion acquisition, precursor MS1 scan (*m*/*z* 355–1700) was acquired in the Orbitrap at a resolution setting of 120,000, followed by Orbitrap HCD-MS/MS and ETD-MS/MS of the five most abundant multiply charged precursors in the MS1 spectrum; a minimum MS1 signal threshold of 10,000 ions was used for triggering data-dependent fragmentation events; MS2 spectra were acquired at a resolution of 30,000. Data processing was carried out using Proteome Discoverer 1.4 software (Thermo Fisher Scientific) as previously described (21) with minor modifications as outlined below. Raw data files (.raw) were processed using the Sequest HT node and searched against the canonical *S. cerevisiae* proteome (7225 entries) downloaded from the UniprotKB (http://www.uniprot.org/; October, 2013). In all cases, the precursor mass tolerance was set to 10 ppm and fragment ion mass tolerance to 0.05 Da. Carbamidomethylation on Cys was used as a fixed modification, oxidation of Met, deamidation of Asn and hexosylation of Ser and Thr were used as variable modifications. A maximum of eight variable modifications were allowed per peptide. A maximum of two missed cleavage sites were tolerated. Spectral assignments at the medium confidence level (*p* > 0.01) and below were resubmitted to a second Sequest HT node using semispecific trypsin, chymotrypsin, or GluC proteolytic cleavage. Final results were filtered for high-confidence (*p* < 0.01) identifications only. Peptide confidence levels were calculated using the Target Decoy PSM Validator node of Proteome Discoverer 1.4. HCD spectra were further processed with a subtraction routine as previously described (23). Briefly, all HCD spectra were extracted to a separate .mgf file and the exact masses of one to four hexose residues were subtracted from each precursor ion resulting in four separate .mgf files. Each .mgf file was subsequently processes as described above with the exception of omitting hexose as variable modification at Ser or Thr residues.

MS analyses of (glyco-)peptides from enriched covalently linked cell wall proteins were performed on a hybrid Velos LTQ Orbitrap mass spectrometer (Thermo Scientific) equipped with an ETD unit and coupled to an Eksigent-nano-HPLC system (Eksigent Technologies, Dublin, CA). Separation of peptides was done on a self-made column (75 μm × 80 mm) packed with C18 AQ 3 μm resin (Bischoff Chromatography, Leonberg, Germany). Peptides were eluted with a linear gradient from 2–31% acetonitrile in 53 min at a flow rate of 250 nL/min. For ETD measurements, full MS data were acquired in the Orbitrap unit in a mass range of 300–1700 *m*/*z*, with an automatic gain control (AGC) setting of 1 × 10^6^, at a resolution of 60,000 at 400 *m*/*z* and a maximum injection time of 250 ms. ETD-MS/MS spectra were acquired in the data dependent mode with up to 10 ETD spectra recorded in the linear ion trap (50 ms injection time). A minimal signal threshold of 1000 was required to trigger the MS/MS acquisition. Supplemental activation energy was activated, and the AGC value was set at 5 × 10^4^. Fluoranthene was used as anion with an AGC value of 1 × 10^5^ and a reaction time of 100 ms. For HCD measurements, full MS scans were done at a resolution of 30,000 at 400 *m*/*z* (250 ms injection time). A maximum of 10 HCD MS/MS scans were acquired with normalized collision energy set to 40%, enabling the collision energy to be stepped (width 15%, three steps). Fragment ions were detected in the Orbitrap at a resolution of 7500 at 400 *m*/*z* (200 ms injection time). All measurements were performed with one microscan with the exception of the LysN ETD measurement with two microscans for the MS/MS acquisition. The Mascot Distiller v2.5 (Matrix Science Inc.) was used to convert MS and MS/MS spectra in Mascot generic format (mgf). For all measurements, MS/MS spectra were searched against the *S. cerevisiae* in-house protein database including common contaminants (fgcz_4932; sequences based on the Uniprot *S. cerevisiae* reference proteome 559292) using the Mascot search algorithm v2.4 (Matrix Science Inc.) with the following parameters: Carbamidomethylation (Cys) as fixed modification; oxidation (Met), hexose (Ser), hexose (Thr) as variable modifications for ETD measurements and neutral loss of hexose for HCD measurements in addition to oxidation of methionine. Further, a peptide tolerance of 5 ppm and a fragment ion tolerance of 0.8 Da or 0.02 Da were used for ETD or HCD, respectively. LysC, LysN, or AspN were set as proteases with a maximum of three missed cleavages. The maximum false discovery rate was set at 1% and peptides with an e-value of above 0.05 were rejected. All acquired data were manually verified using the Mascot search output and the Xcalibur software v2.0 (Thermo Scientific).

##### Data Analysis and Bioinformatics

Gene Ontology (GO) term analysis was performed using the Database for Annotation, Visualization, and Integrated Discovery (DAVID, v6.7; (25)). Subcellular localization was additionally analyzed based on manually curated information from the Saccharomyces Genome Database (SGD; (26)) and high-confidence cellular component GO terms extracted from the Compartments database ((27); April, 2015). GO terms of interest were filtered manually: wherever possible child terms that refer to extrinsic parts of membranes were excluded from membrane-enclosed compartments, and integral or intrinsic parts of membrane parts were counted toward the membrane-enclosed compartments. Proteins that did not match the criteria and could not be assigned to a specific subcellular localization were regarded as not annotated (NA).

Sequence analysis was performed using an in-house prepared “R” script, which is available upon request. For sliding window analysis a default window size of 21 amino acids were chosen. This way, the Ser/Thr-content, the hydropathy (according to Kyle & Doolittle, (28)), the FoldIndex(c) (http://bip.weizmann.ac.il/fldbin/findex, (29)) were calculated for every *O*-Man site of the secretome. Secondary structure probabilities were predicted externally using NetSurfP 1.1 (http://www.cbs.dtu.dk/services/NetSurfP/, (30)). The corresponding sequence windows were used for single-sided (WebLogo 3.4, http://weblogo.threeplusone.com) and two-sided Logo-plots (www.twosamplelogo.org), respectively (31, 32). Two-sample Logo-plots were tested against all Ser/Thr positions of the secretome as a reference with a threshold of *p* < 0.01.

##### Nonreductive β-Elimination and HPEAC-PAD

*O*-Man glycans were released from tryptic peptides of total cell lysates by nonreductive β-elimination according to Zheng *et al.* (33), and separated from the peptides by solid phase extraction (Sep-Pak C18, Waters). High-performance anion-exchange liquid chromatography with pulsed amperometric detection (HPEAC-PAD) was performed according to the literature (34), using a CarboPac PA1 column connected to the ICS-3000 system (Dionex, Germering, Germany) and quantified by PAD. The column was equilibrated with five column volumes of solvent A (ultra-pure H_2_O) at a flow rate of 1 ml min^−1^. Column temperature was kept contant at 25 °C. Baseline separation of carbohydrates was achieved by increasing the concentration of solvent B (300 mm NaOH) in solvent A as follows: From 0 to 25 min 7.4% B, followed by a gradient to 100% B within 12 min, hold for 8 min at 100% B, return to 7.4% B and equilibration of the column for 12 min. Data acquisition and quantification was performed with Chromeleon 6.7 (Dionex).

##### Western Blot Analysis of Total Cell Lysates

Total cell lysates were prepared in ice-cold 1 × TBS buffer including 1 mm PMSF and 1 mg/ml pepstatin. Proteins were analyzed by Western blot as previously described (18). Polyclonal anti-Hsp150 (35) was used at a dilution of 1:5000, and anti-Kex2 (gift of W. Tanner) at a dilution of 1:1000.

##### Cell Free Microsomal Translation/Translocation Assay

*In vitro* protein translation and translocation into yeast microsomes (strain SEY6210) was performed as described recently (8). Proteins were separated on 15% polyacrylamide gels and detected by Western blot using the anti-FLAG (Sigma-Aldrich, Taufkirchen, Germany) monoclonal antibodies at a dilution of 1:10,000. For Endo H treatment microsomes (24 μg) were purified, resuspended in denaturing buffer and heated at 65 °C for 15 min. Thereafter, 250 U of Endo H (New England Biolabs) were added and samples incubated at 37 °C for 1 h in the presence of 5 mm PMSF. Experimental details are outlined in the supplemental Experimental Procedures.

##### Experimental Design and Statistical Rationale

In total, eight MS/MS datasets were recorded for enriched *O*-Man glycoproteins from WT and *KTR*Δ cells, each comprising results from data processing of HCD- and ETD activation types as well as results from processed spectra accounting neutral loss of one to five hexoses. Digests of *KTR*Δ cells were performed in duplicate, so that two datasets each originated from trypsin, GluC, and chymotrypsin digestion. A single dataset originated from tryptic digests of WT cells and were included for analysis because the results were found in strong accordance with the results obtained from *KTR*Δ cells. Microsomal translation/translocation assay, and glycan profiling using HPEAC-PAD were performed at least in biological triplicates.

## RESULTS

### 

#### 

##### LWAC Enrichment of O-Man Glycopeptides Identified 511 Glycoproteins and Over 2600 O-Glycosylation Sites

**Fig. 1.**
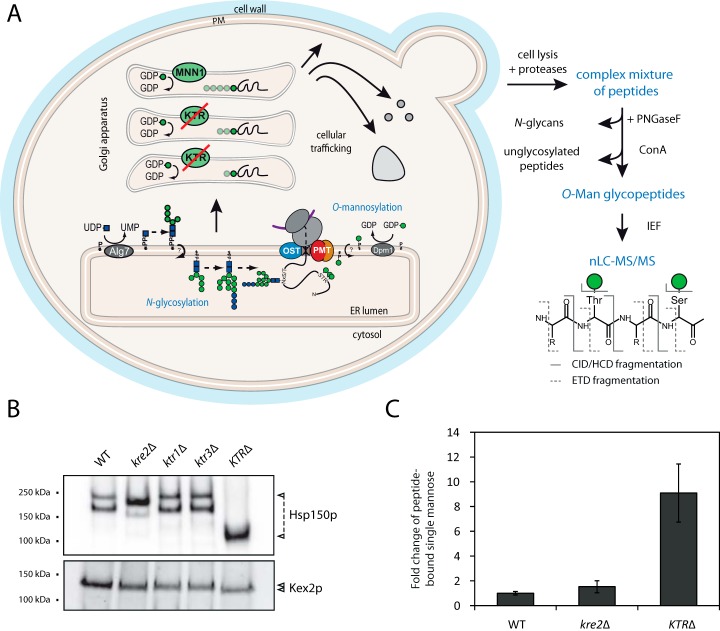
**The *O-*Man glycoproteomics strategy on yeast mutant *KTR*Δ.** *A*, Illustration of the *O*-Man glycosylation pathway and the workflow for enrichment and analysis of *O*-Man glycopeptides by Con A lectin chromatography and HCD/ETD-MS/MS. *B*, *C*, Simplified *O*-Man glycans in the mutant strain *KTR*Δ. *B*, Western blot analysis of the *O*-mannosylated targets Hsp150 and Kex2. 30 μg of total cell lysates were separated by SDS-PAGE and analyzed by Western blot using protein-specific antibodies as indicated (see Experimental Procedures). Block of glycan elongation is monitored by electrophoretic mobility shift in WT and mutant strains as indicated. *C*, Relative quantitation of peptide-bound single mannose in WT and indicated strains by HPEAC-PAD. Mean values of three biological replicates ± standard deviation are shown.

We set out to map the *O*-Man glycoproteome in baker's yeast using our recently developed shotgun LWAC enrichment approach and HCD/ETD-based MS/MS. In addition to WT yeast, we used a mutant strain lacking the three major Golgi-located α1,2-mannosyltransferases Kre2, Ktr1, and Ktr3 (9) in order to reduce the sample complexity of peptides bearing glycan chains of heterogeneous length (Fig. 1*A*). In the *kre2*Δ*ktr1*Δ*ktr3*Δ (*KTR*Δ) mutant the elongation of *O*-linked mannoses in the Golgi apparatus is blocked to a large extent when compared with the WT strain and the single mutants (Fig. 1*B*, 1*C*; (36)). As an example for the simplification of the *O*-Man glycans in this mutant, the electrophoretic mobility shift of the known highly *O*-mannosylated, secreted heat shock protein Hsp150, and the moderately *O*-mannosylated Golgi-resident protease Kex2 (37) are shown in Fig. 1*B*. In addition, the relative quantification of peptide-bound single mannose residues from tryptic digests (for details see Experimental Procedures) by HPEAC-PAD further showed the substantial reduction of the heterogeneity of glycan chain length in this mutant (Fig. 1*C*).

Total cell lysates from the WT strain and the *KTR*Δ mutant were digested with the endoproteinases trypsin, GluC, or chymotrypsin. *N*-linked glycans were removed and *O*-Man glycopeptides enriched on the lectin Con A. After additional fractionating, peptides were analyzed by HCD/ETD-MS/MS (Fig. 1*A*). This way, we identified ∼2600 *O*-mannosylation sites in 396 unique proteins. Approximately 2300 of these sites were assigned from ETD peptide spectra (supplemental Table S2 and supplemental Fig. S1). Further, 880 sites were assigned from HCD-MS/MS. Taking into account that HCD-MS/MS spectra suffer from neutral loss of hexose residues because of the specific mechanisms of fragmentation and that residual glycan elongation can be observed, these sites are considered to be ambiguous as long as the modification sites were not confirmed by ETD-MS/MS. Additional 115 *O*-mannosylated proteins were identified based on a hexose subtraction routine from HCD spectra only, yet glycosylation sites could not be assigned (detailed under Experimental Procedures; (21)). All glycopeptides identified with the shotgun approach are available in supplemental Table S3. For each protein the number and position of *O*-Man sites were extracted from sequence alignments. In the context of the yeast proteome, these results are available in supplemental Table S2. Additional information, such as the classification of proteins known or predicted to be translocated into the ER (hereinafter referred to as the yeast secretome) according to Ast *et al.* (38), the number and position of known *N*-glycosylation sites determined by Zielinska *et al.* (39), the subcellular localization and membrane assignment, as well as further information compiled from SGD (http://www.yeastgenome.org/) and the UniProt Knowledgebase (UniProtKB, http://www.uniprot.org/), is included in supplemental Table S2.

**Fig. 2.**
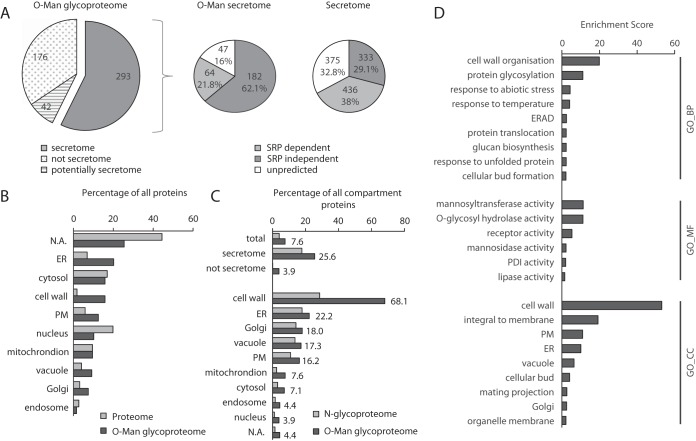
**The yeast *O-*Man glycoproteome.** *A*, 511 *O*-Man glycoproteins from all subcellular compartments were identified by HCD/ETD-MS/MS (left pie chart). Among 1144 proteins targeted to the ER (right pie chart; extracted from Ast *et al.* (38) and referred herein as secretome), 293 *O*-Man glycoproteins (left pie chart) could been assigned. Within this fraction, SRP-independently translocating proteins are specifically enriched (middle pie chart) (also see supplemental Table S2) *B*, Proteins of the cell wall, the plasma membrane (PM) and the secretory pathway are enriched in the *O*-Man glycoproteome. The percentage of proteins from the corresponding subcellular compartments in the total yeast proteome (light gray) and the *O*-Man glycoproteome (dark gray) are shown. Higher values in the *O*-Man glycoproteome indicate an overrepresentation of proteins from a specific compartment in relation to the yeast proteome and vice versa. *C*, Coverage of proteins from distinct subcellular compartments in the *O*-Man glycoproteome. The percentage of proteins from different subcellular compartments identified within the *O*-Man glycoproteome (dark gray) are shown. For comparison, the distribution of recently described *N*-linked glycoproteins of *S. cerevisiae* is depicted (light gray) (39). *B*, *C*, Annotations are based on manual curation from SGD and selected high-confidence cellular compartment GO terms from the Compartments database. Proteins without any annotation according to these criteria are summarized as not annotated (NA). The corresponding listings of proteins including classification of subcellular localization can be found in supplemental Table S2. *D*, GO term enrichment analysis using DAVID (v6.7). GO term enrichment was performed on all identified *O*-Man glycoproteome Uniprot accessions (extracted from supplemental Table S3) using GO FAT terms, an EASE score of 0.1, and the *S. cerevisiae* reference list. Enrichment scores were extracted for the respective GO term clusters and clusters were named by representative terms. Biological process (GO_BP), molecular function (GO_MF) and cellular compartment (GO_CC) are shown.

Analysis of the 511 identified *O*-Man glycoproteins showed that a total of 293 classified as glycoproteins from the yeast secretome (Fig. 2*A*, (38)). Manual inspection revealed a minimum of 10 proteins that we consider part of the secretome (*e.g.* members of the cell wall-localized seripauperin family), and 32 uncharacterized gene products of unknown localization and function, reducing the number of nonsecretome *O*-Man glycoproteins to a total of 176 (Fig. 2*A*). These proteins originate from mitochondria, the cytoplasm or the nucleus, and are not exposed to the PMT *O*-mannosylation machinery in the ER. We propose that the biosynthesis, regulation, and function are different from the classical *O*-Man glycoproteins entering the secretory pathway. Nucleocytoplasmic proteins that have been identified in a subset of the experiments presented in this study, are highlighted in supplemental Tables S2 and S3, and are described separately (22).

Here, we focus only on the 293 secretome proteins containing ∼2200 *O*-Man sites (supplemental Table S2), which are targets of PMT-based *O*-mannosylation. With regard to the classification of the yeast secretome by Ast *et al.* (38), we found that 26% of the proteins of the yeast secretome contain *O*-Man glycans. A considerable fraction of these glycoproteins (62%, 182 out of 293) enter the ER in a signal recognition particle (SRP) -independent manner (Fig. 2*A*). This fraction of cellular components is known to be enriched for proteins with glycosylphosphatidylinositol (GPI) -anchoring sequences, and indeed, we identified 78% (45 out of 58) of all known or predicted GPI-anchored proteins to be *O*-mannosylated (supplemental Table S2). When compared with the yeast proteome, the proportion of cell wall and plasma membrane proteins is decidedly enriched in the total *O*-Man glycoproteome. In addition, a large fraction of proteins from the vacuole and the endomembrane system was found, particularly proteins from the ER (Fig. 2*B*, 2*C*). Cellular compartments were confirmed performing an automated GO enrichment analysis using the DAVID database (Fig. 2*D*, GO_CC). In detail, we assigned *O*-Man glycans to 68% of all proteins that are annotated for cell wall localization. Furthermore, 22% of the ER-localized, 18% of the Golgi-resident, 17% of the vacuolar, and 16% of all plasma membrane annotated proteins are targets of *O*-mannosylation (Fig. 2*C*). For the vast majority of these proteins this post-translational modification is described for the very first time. When comparing with the yeast *N*-glycoproteome reported by Zielinska *et al.* (39), we find that 56% (152 out of 272) of the *N*-glycoproteins also contain *O*-Man glycans (supplemental Table S2). Although the overall ratios between *N*-glycosylated and *O*-mannosylated proteins of the secretory pathway are similar, we found a notably higher amount of *O*-Man containing cell wall proteins (Fig. 2*C*). All glycoproteins and their corresponding classifications used for these analyses are included in supplemental Table S2.

**Fig. 3.**
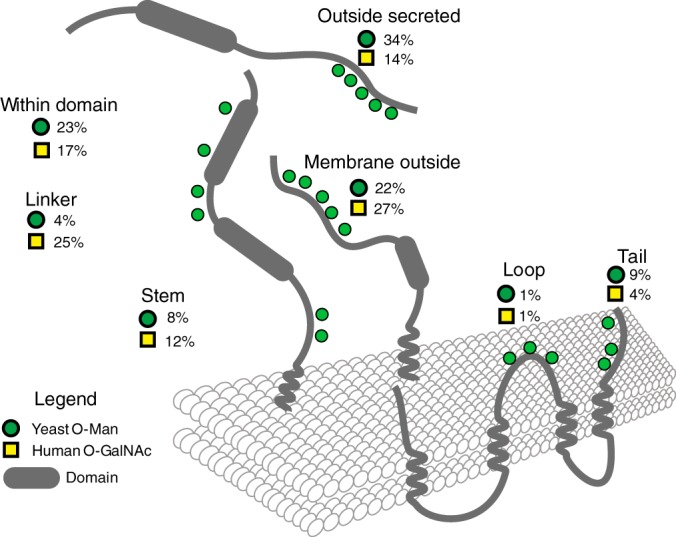
**Organisation of *O-*Man sites in protein domains in comparison with *O-*GalNAc.** *O-*Man and *O*-GalNAc sites were compared between yeast and human to understand the overall distribution of glycosylation on proteins. Transmembrane information was retrieved from UniProt (Oktober, 2015), and domain information retrieved from Interpro 53.0. Using the “R” package Rgator, sites were classified into different regions on the protein (glycodomains), and summaries of the numbers produced.

We classified the sites of glycosylation into domains of proteins using the GlycoDomainViewer. This method has previously been used to better understand the mammalian *O*-GalNAc glycoproteome (40) and can be used to compare general properties of the two glyco-proteomes (see Discussion). The method functions by classifying sites based upon their position on the protein with respect to conserved folds. It allows for extraction of glycosylated domains, stem, linker, and multipass transmembrane loop and tail regions. As shown in Fig. 3, the major glycodomain classifications places yeast *O*-Man sites outside of protein folds (56% of sites), but not in linker (4%) or stem (8%) regions. The sites on proteins lacking a transmembrane domain make up the largest proportion of these sites (34%). The next largest glycodomain set is the 23% of the sites found within domains on proteins. The most common glycosylated domains are glycoside hydrolase related domains (IPR013781 and IPR017853, mostly Scw4, Scw10, and Gas1), the MIR motif found on PMTs (IPR016093) and the thioredoxin fold as found on protein disulfide isomerase (PDI) family members (IPR012336, see below). The data are available online to explore on the GlycoDomainViewer (http://glycodomain.glycomics.ku.dk/doi/10.1074/mcp.M115.057505/), where glycosites are viewed in the context of predicted domain structures of proteins.

##### Cell Wall Proteins Are Major Targets of O-Mannosylation

Our shotgun LWAC approach identified 82 unique cell wall and cell wall-associated proteins for which a total of ∼1400 (∼63%) *O*-Man glycosylation sites were assigned (supplemental Table S2). Accordingly, proteins involved in cell wall organization, protein glycosylation and abiotic stress response, especially glycosyltransferase, -hydrolase, and receptor activities, are significantly enriched in the *O*-Man glycoproteome as can be seen by GO enrichment analyses (Fig. 2*D*, GO_BP and GO_MF). Some of the cell wall proteins are extensively mannosylated, as illustrated by the occurrence of 1122 *O*-Man sites in only 32 proteins, including among others structural cell wall proteins (*e.g.* Hsp150: 76 *O*-Man sites) as well as glycosyltransferases and -hydrolases (*e.g.* Gas1: 52 *O*-Man sites; Egt2: 48 *O*-Man sites) (supplemental Table S2).

Using an independent approach targeting cell wall proteins only, but omitting the LWAC enrichment of glycopeptides, we validated *O*-mannosylation for a subset of the cell wall proteins. Thereto, we isolated cell wall fractions from WT yeast that were enriched for proteins covalently linked to the cell wall glucan (24). After removal of *N*-linked glycans by treatment with endoglycosidase H, peptides were prepared using the endoproteinases LysC, LysN, or ArgN. Then, *O*-Man glycans of heterogenous length were trimmed with Jack bean α-mannosidase, and the resulting (glyco)peptides analyzed without further enrichment by HCD/ETD-MS/MS. We identified a total of 107 *O*-Man peptides from 24 cell wall proteins (supplemental Table S4), which largely overlap with the tryptic glycopeptides found in the total cell lysates from the *KTR*Δ mutant (examples are shown in supplemental Fig. S2). In both types of experiments, a considerable coverage of all potential generated peptides that are applicable for MS/MS analysis (*i.e.* length of 6 to 25 amino acids) was achieved (supplemental Fig. S2), all in all showing the sensitivity and robustness of our shotgun LWAC-based method.

##### O-Man Sites Found Widely on ER and Golgi Resident Proteins

Although it was expected that cell wall proteins are major carriers of *O*-Man glycans, it was somewhat surprising to find that a significant number of *O*-Man glycosites were distributed on numerous proteins of the secretory pathway. Despite few exceptions, *O*-mannosylation of these proteins had not been considered yet. ER proteins represent the second largest group of proteins in the *O*-Man glycoproteome (Fig. 2*B*–2*D*). For nine of the 103 ER-annotated proteins, ten or more *O*-Man sites could be mapped (supplemental Table S2). Among those glycoproteins are Sed4 (55 *O*-Man sites) and its paralog Sec12 (15 *O*-Man sites), which are guanine nucleotide exchange factors required for the initiation of COPII vesicle formation in ER to Golgi transport (41); Slp1 (21 *O*-Man sites), an integral ER membrane protein with a suggested role in folding of membrane proteins (42); and the Hsp70-like chaperone Lhs1 (15 *O*-Man sites) that acts with the ER luminal Hsp70 Kar2 during protein import into the ER (43). For the majority of ER proteins however, less than six *O*-Man sites have been mapped (supplemental Table S2). In addition, especially proteins were found *O*-mannosylated that play crucial roles in biological processes and molecular functions associated with the ER, such as protein trafficking (*e.g.* Sec12, Sec20), glycosylation (*e.g.* Pmt1, Ost1, Wbp1), protein folding and quality control (*e.g.* Lhs1, Kar2, Pdi1, Rot1) as well as response to ER stress conditions (*e.g.* Ire1) (Fig. 2*D*, GO_BP, GO_MF). Selected examples are shown in Table I.

**Table I:** Selected ER-localized O-Man glycoproteins involved in glycosylation, ER quality control and intracellular trafficking

Function	Identifiers		Essential	Number of sites		Orthologs in human
	Locus	Name		*N-*glyco (confirmed)	*O-*Man (total)	
Glycosylation: *N-*glyco	YJL002C	OST1	YES	4	7	GalNAc
Glycosylation: *N-*glyco	YEL002C	WBP1		2	6	
Glycosylation: *N-*glyco	YMR149W	SWP1			6	
Glycosylation: *O-*Man	YAL023C	PMT2		1	13	
Glycosylation: *O-*Man	YDL095W	PMT1		3	11	
Glycosylation: *O-*Man	YGR199W	PMT6		4	3	
Glycosylation: *O-*Man	YJR143C	PMT4			1	
Glycosylation: *O-*Man	YOR321W	PMT3		1	1	
Quality Control: ERAD	YNL008C	ASI3		1	1	
Quality Control: Folding	YOR154W	SLP1		3	21	
Quality Control: HSP	YKL073W	LHS1		10	15	GalNAc
Quality Control: HSP	YMR200W	ROT1	YES	3	13	
Quality Control: HSP	YJL034W	KAR2	YES		8	GalNAc
Quality Control: HSP	YOL031C	SIL1			7	
Quality Control: HSP	YMR214W	SCJ1			6	
Quality Control: HSP	YJL073W	JEM1		1	1	GalNAc
Quality Control: *N-*glyco	YDR057W	YOS9		1	11	GalNAc
Quality Control: *N-*glyco	YAL058W	CNE1		4	4	
Quality Control: PDI	YML130C	ERO1	YES	6	11	
Quality Control: PDI	YDR518W	EUG1		2	9	GalNAc/Man
Quality Control: PDI	YCL043C	PDI1	YES	7	6	GalNAc/Man
Quality Control: PDI	YIL005W	EPS1		2	5	GalNAc/Man
Quality Control: PDI	YOR288C	MPD1		2	2	GalNAc/Man
Quality Control: UPR	YHR079C	IRE1			5	
Trafficking: COPI	YDR498C	SEC20	YES		5	
Trafficking: COPII	YCR067C	SED4			55	
Trafficking: COPII	YNR026C	SEC12	YES	1	15	
Trafficking: COPII/p24	YGL200C	EMP24			4	GalNAc
Trafficking: COPII	YIL039W	TED1		3	2	
Trafficking: COPII/p24	YML012W	ERV25			2	GalNAc
Trafficking: COPII/p24	YDL018C	ERP3			2	GalNAc
Trafficking: COPII	YAL042W	ERV46			1	
Trafficking: COPII/p24	YAR002C-A	ERP1			1	

In addition to ER proteins, *O*-Man sites could be assigned to a total of 38 Golgi-located proteins. Particularly remarkable is that 13 of these proteins directly function in outer chain elongation of *N*-linked glycans. We identified all but two of the mannosyltransferases involved in this process (Och1; M-PolI: Mnn1 and Van1; M-PolII: Mnn9, Mnn11 and Hoc1; Mnn2, Mnn5, Kre2, Ktr4), the GlcNAc-transferase Gnt1, and the proteins involved in the transport of GDP-Man into the Golgi lumen (Vrg4, Gda1) (supplemental Table S2).

**Fig. 4.**
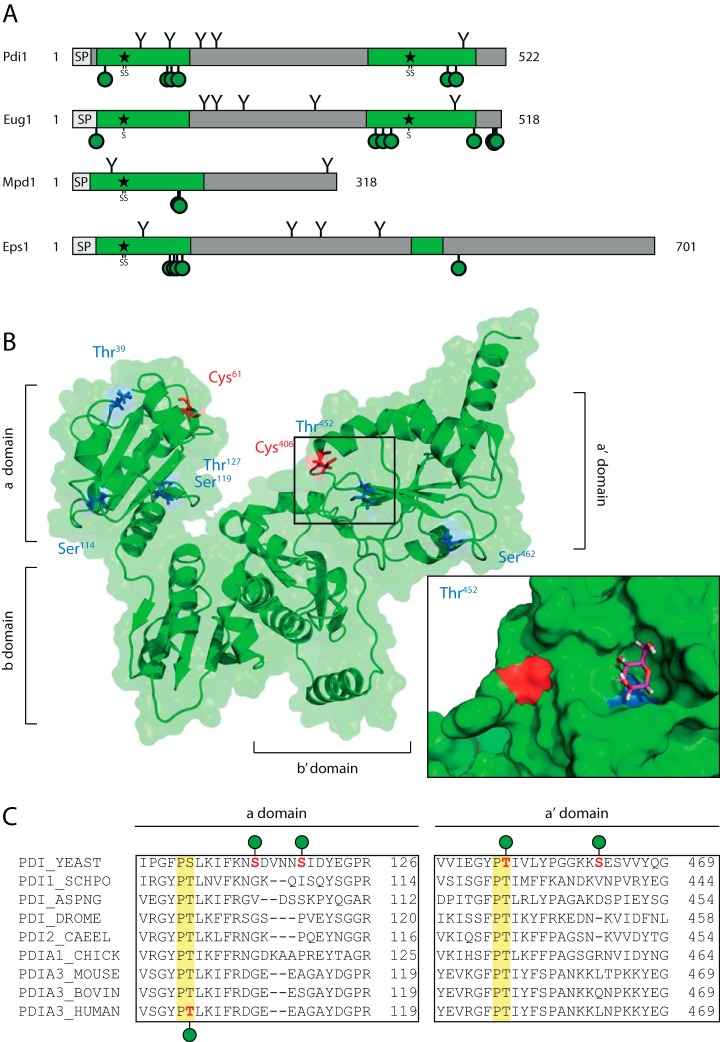
***O*-Mannosylation of Pdi1 and PDI-like proteins.** *A*, Schematic illustration of structural domains from Pdi1 and the PDI-like ER proteins Eug1, Mpd1 and Eps1 from baker's yeast. Active sites (stars) and thioredoxin domains (green) are highlighted. Putative *N*-linked glycosylation sites (Y shapes) are indicated as well as all *O*-Man sites (green circles) identified. *O*-Man sites are generally clustered in specific regions of the thioredoxin domains, flanking the active sites and the hydrophobic core region. *B*, Ribbon diagram and transparent surface representation of Pdi1 (PDB: 2b5e) with indicated a, b, b', and a' domains. Reactive sites of the a and a' domain, Cys^61^ and Cys^406^, are indicated in red. Mapped *O*-Man sites are indicated in blue. Highlighted excerpt is featuring the active site Cys^406^ next to the *O*-Man site Thr^452^ as indicated. For representation, a mannose residue was modeled in the diagram and manually fitted for attachment and orientation using Pymol. *C*, Multiple sequence alignment of the corresponding regions in Pdi1 homologs from fungi and mammalian origin.

Intriguingly, many of the ER and Golgi located proteins are orthologous to human proteins. For a total of 3571 yeast proteins, human orthologs with reasonable sequence similarity can be defined (based on information from SGD). 79 of these proteins are *O*-glycosylated in both human and yeast. Here again, these proteins mainly account for few biological processes and molecular functions, namely the involvement in ER quality control and folding, protein sorting and vesicular transport, and protein disulfide isomerase, phosphatase or peptidase activities (examples in Table I and supplemental Fig. S3). When aligning these proteins, we found that in general, regions of glycosylation are conserved, and in few cases even individual sites are shared by *O*-Man in yeast, and *O*-GalNAc and/or *O*-Man in humans (supplemental Fig. S3). An example for conserved *O*-glycosylation is the PDI family. For the ER luminal PDI – Pdi1 – and three other ER-localized PDI-like proteins – Eug1, Mpd1, and Eps1 – *O*-Man glycans could be specifically assigned. All sites are present in the domains containing the active site with a CysXXCys motif (Fig. 4*A*). In Pdi1, one of these sites (Thr^452^) is placed in a highly-conserved ProThr-motif (Fig. 4*C*). An inspection of the recombinant yeast Pdi1 crystal structure revealed that this motif is directly adjacent to one of the catalytic cysteine residues (Cys^406^) (Fig. 4*B*; (44, 45)). Molecular modeling showed that at position Thr^452^ an *O*-linked mannose can be fitted precisely into a prominent groove at the protein surface (Fig. 4*B*, detail enlargement). *O*-Man glycans are also present in the conserved ProThr-motif of the mammalian Pdi1-homolog PDIA3 from human breast cancer cells (21), making Pdi1 a striking example that emphasizes the relevance of our findings.

##### O-Mannosylation is Favored in Unstructured Regions and β-Strands

**Fig. 5.**
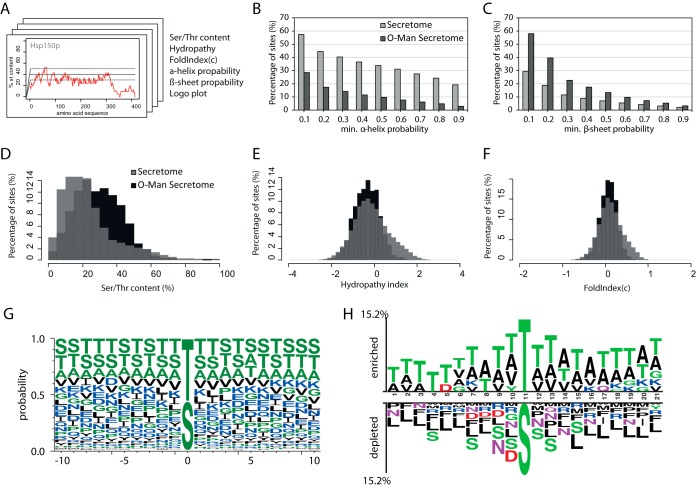
**Analysis of sequential and structural features of *O-*Man sites.** *A*, *O*-Man sites were analyzed using an in-house prepared “R” script performing sliding window analyses on protein sequences. The default sequence window size was 21 amino acids, ten amino acids *N*- and *C*-terminal of the respective site of interest. An example of a sliding window analysis result measuring the Ser/Thr content in percent is shown for Hsp150. Values calculated this way include the general Ser/Thr content in percent, the hydropathy, and the FoldIndex(c) as a measure for structural disorder. Additionally, values for the probability for α-helices and β-strand secondary structures were calculated externally using NetSurfP 1.1. Positions of *O*-Man modifications were extracted from supplemental Table S3. The corresponding values were plotted in histograms and bar plots, and the corresponding sequence windows were used for generation of Logo plots. In each case, only proteins and site information from proteins entering the secretory pathway (according to Ast *et al.* (38)) were taken into account. *B*, *C*, Bar plot summarizing the results of *B*, α-helix and *C*, β-strand prediction using NetSurfP 1.1. Bars represent the number of *O*-Man sites (dark gray) in percent that are located in protein regions with a minimum secondary structure probability as indicated. *O*-Man sites were compared with the distribution of all Ser/Thr sites from proteins entering the secretory pathway (light gray). *O*-Man sites were found underrepresented in regions of high α-helices, but overrepresented in regions of high β-strand probability. *D–F*, Histograms show the results of sliding window calculations of *D*, the general Ser/Thr content, *E*, the hydropathy, and *F*, the FoldIndex(c) surrounding the *O*-Man sites. *O*-Man sites (black) were compared with the distribution of all Ser/Thr positions in the secretome (gray). *O*-Man sites were found to be overrepresented in regions of 30–50% Ser/Thr content. In addition to that, *O*-Man sites are situated in hydrophilic and intrinsically disordered protein regions. *G*, One-sided Logo plot of analyzed sequence windows (using WebLogo 3.4). Size of the characters indicates the occurrence of amino acids within the sequence windows surrounding the *O*-Man site. Amino acids are colored according to their hydrophobicity: hydrophilic amino acids are blue, neutral amino acids are green, and hydrophobic amino acids are black. *H*, Two-sided Logo-plot of analyzed sequence windows (using Two Sample Logo). Sequence windows of *O*-Man sites were compared against the sequence windows of all Ser/Thr positions in the secretome. A threshold for enrichment and depletion of *p* > 0.01 was used. Amino acids are colored according to their chemical properties: polar amino acids are depicted in green, neutral amino acids are purple, basic amino acids are blue, acidic amino acids are red, and hydrophobic amino acids are black.

To gain further insight into the molecular features of target proteins defining PMT-based *O*-mannosylation, we analyzed the peptide sequences surrounding the determined *O*-Man sites in a sliding window analysis (Fig. 5*A*). This way, for every Ser/Thr and every *O*-Man site in the secretome the general Ser/Thr-content surrounding the site of interest, the immediate hydropathy, the FoldIndex(c) as a measure for structural disorder, and the probability for α-helical and β-strand secondary structures (NetSurfP 1.1) were calculated. These analyses revealed that the majority of *O*-Man sites are preferentially placed within regions of a Ser/Thr-content of at least 20 to 50% (∼4 to 10 Ser or Thr residues in close proximity) (Fig. 5*D*; for individual examples see supplemental Fig. S2). We not only experimentally confirmed the predictions made, but also found that glycosylation of Thr is favored over Ser, and that apart from hydroxyl-amino acids, Ala and Val residues are preferred in the proximity of the *O*-Man sites (Fig. 5*G*, 5*H*). In contrast, Leu and Asp residues are less favored, the latter especially at positions −4 to −1. Although we did not identify evident sequence features suggestive of a glycosylation motif, we found that disordered protein characteristics favor the addition of *O*-Man glycans (Fig. 5*F*). Furthermore, *O*-Man sites are underrepresented in regions with high α-helix probability, but overrepresented in β-strand folds (Fig. 5*B*, 5*C*).

##### O-Mannosylation is Impeded by N-Glycosylation

In *S. cerevisiae*, numerous proteins contain both, *N*-linked and *O*-Man glycans, and our previous work revealed that these types of glycosylation can even compete for some acceptor proteins (8, 13). *N*-glycosylation is initiated at the ER by the oligosaccharyl transferase (OST) complex, which catalyzes the transfer of the oligosaccharide GlcNAc_2_Man_9_Glc_3_ from a lipid-linked donor to Asn residues of the acceptor sequence Asn-X-Ser/Thr. As shown in Fig. 5*H*, Asn residues are significantly under-represented in the direct vicinity of *O*-Man sites. Most frequently Asn is absent at the −2 position of the *O*-Man site, which clearly reflects the *N*-glycosylation sequon Asn-X-Ser/Thr. *O*-mannosylation of the Ser or Thr in this sequence was only observed for 25 out of the 836 *N*-glycosylated sequons identified by Zielinska *et al.* ((39); supplemental Table S2). Considering the high Ser/Thr content surrounding the *O*-Man sites also the Asn residues in the positions −1 to +3 are likely to represent canonical *N*-glycosylation motifs.

**Fig. 6.**
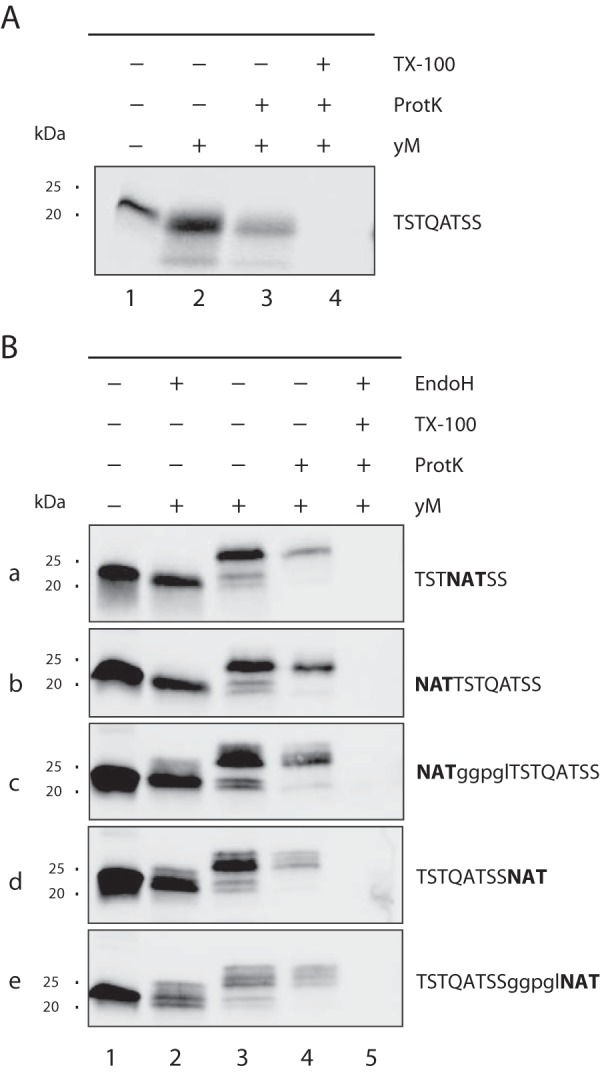
**Context dependent *O-*mannosylation of a model acceptor substrate in *in vitro* translation/translocation/glycosylation assays.** *A*, *B*, *O*- and *N*-linked glycosylation was monitored *in vitro* using a cell free translation/translocation/glycosylation assay described by Loibl *et al.* (8). *A*, Translated proteins and translocation products in the presence of yeast microsomes (yM) of the FLAG-tagged Ccw5-based model substrate TSTQATSS (schematically represented in supplemental Fig. S4) and *B*, the indicated derivatives thereof are shown. Details are outlined under Experimental Procedures. Translocation products were confirmed in the presence of Proteinase K (ProtK) and the detergent Triton X-100 (TX-100). Proteins of purified microsomes were treated with Endo H to remove *N*-linked glycans. Proteins were separated by SDS-PAGE and analyzed by Western blot using anti-FLAG antibodies.

To further address this aspect, we took advantage of a cell-free translation/translocation/glycosylation system that we recently established to study *O*-mannosylation at the ER translocon (8). Briefly, reactions were started by mixing a translation lysate, an mRNA of interest, and yeast WT microsomes. Translocation products inside the microsomes were selected by Proteinase K treatment, resolved by SDS-PAGE and detected by Western blot. We previously showed that in this system, the protein Ccw5 is efficiently decorated with *O*-Man glycans (8, 13). Based on this protein, we designed a model substrate containing a short stretch of Ser/Thr residues (TSSQATSS, supplemental Fig. S4), which are *O*-mannosylated in yeast microsomes, as indicated by the fuzzy appearance of the translocation product shown in Fig. 6*A* (compare lane 1 with lane 2 and 3). *O*-mannosylation of the translocated protein was further confirmed by Con A lectin affinity pull down and LC-MS/MS (supplemental Fig. S4). A sequon sequence centered in or *N*-terminal of the Ser/Thr-stretch was predominantly *N*-glycosylated (Fig. 6*B*; Endo H treatment, compare in panel a or b, lanes 3 and 4 with lane 2), leading to a significant reduction of *O*-mannosylation of the neighboring Ser/Thr residues (Fig. 6*A*, 6*B*; compare lane A2 with lane B2 in panel a and b). In agreement with our glycoproteome data, increasing the distance between the sequon and the Ser/Thr-stretch partly restored *O*-mannosylation (Fig. 6*B*; panel c). Placing the *N*-glycan acceptor site *C*-terminally to the hydroxyl amino acids, we observed the same result although the effect was less pronounced (Fig. 6*B*; panels d and e). The microsomal data are in good agreement with our proteome-wide findings that *O*-mannosylation is less frequent at and in the close proximity of *N*-glycan acceptor sites.

## DISCUSSION

This study presents a comprehensive map of the yeast *O*-Man glycoproteome illustrating that *O*-Man glycosylation affects a wide range of proteins from all major subcellular compartments. Here, we only focused on the *O*-Man glycoproteome of the secretome. We found that at least 26% of the proteins that enter the secretory pathway (a total of 293 proteins) bear *O*-Man glycans. For the vast majority of these glycoproteins *O*-mannosylation has not been shown before, including more than 30 previously uncharacterized proteins of the cell wall and the secretory pathway (supplemental Table S2).

### 

#### 

##### Cell Wall O-Man Glycoproteins

The yeast cell wall is an essential structure that is made of β-glucan (∼50%), mannoproteins (∼50%), and chitin (∼2%) (46). Mannoproteins were found to be *N*-glycosylated, *O*-mannosylated, or both. Conditional *pmt* deletion mutants show various cell wall-related phenotypes (2, 47), and inhibition of *O*-mannosylation results in activation of cell wall integrity signaling pathways (48), emphasizing the crucial role of this modification for the biosynthesis and the maintenance of a functional cell wall. Accordingly, in the *O*-Man glycoproteome proteins involved in cell wall organization and stress response are clearly overrepresented (Fig. 2*D*). In particular, we assigned *O*-Man glycans to 78% of all known or predicted GPI-anchored proteins, which represent more than half of all cell wall annotated proteins (supplemental Table S2). In relation to the yeast *N*-glycoproteome described by Zielinska *et al.* (39), we found a similar proportion of ER and Golgi glycoproteins, but identified a considerably higher number of cell wall proteins (Fig. 2*C*).

We identified about 1400 *O*-mannosylation sites compared with 225 confirmed *N*-glycosylation sites (according to (39); supplemental Table S2). In baker's yeast, about 12% of the protein-linked mannose residues can be released by β-elimination (49). Considering a mean number of ∼3 and ∼120 mannose residues per *O*- and *N*-linked glycan chain, respectively, one can estimate that in average cell wall proteins contain around five to six times more *O*- than *N*-linked glycans. Our data match closely with this prediction, which underscores the quality and validity of the established *O*-Man glycoproteome. Still, the extent of *O*-mannosylation in cell wall proteins is almost certainly underestimated, considering that in some of the Ser/Thr-rich regions of cell wall proteins protease cleavage sites are rare (supplemental Fig. S2, *e.g.* Aga1, Cwp1), and that because of technical constraints detection of highly *O*-glycosylated peptides is limited.

##### ER and Golgi O-Man Glycoproteins

We found that 22% and 18% of all proteins curated for ER and Golgi localization, respectively, are represented in the *O*-Man glycoproteome. *O*-mannosylation has previously been described for a few heavily glycosylated ER proteins, such as Sed4 (41) and Sec12 (50), which were confirmed to be significantly *O*-mannosylated in our study (supplemental Table S2). For the majority of the moderately *O*-mannosylated proteins, this modification has been overlooked so far.

Many proteins involved in *N*-glycosylation, such as the OST subunits Ost1, Wbp1, and Swp1; the trimming glycosidases Cwh41, Mnl1, Mnl2, and Mns1; as well as most of the Golgi mannosyltransferases involved in *N*-glycan processing were found to be *O*-mannosylated. Recent transcriptome analyses of *pmt* mutants identified a compensatory response between *O*- and *N*-glycosylation, which particularly affects the formation of high mannose outer chains (48). In view of this finding, *O*-mannosylation of many of the biosynthetic enzymes of *N*-linked glycans is especially intriguing and might even reflect a regulatory role. Further, *O*-Man glycans could also be assigned to members of the p24 complex involved in the export of GPI-anchored proteins from the ER and the GPI-glycan remodelase Ted1. This finding is specifically significant in the light of the previously reported physical interaction with these proteins and yeast PMTs (51). In addition, numerous proteins with chaperone function such as Lhs1, Kar2, or Rot1; the unfolded protein response receptor Ire1; and proteins involved in the antero- and retrograde ER–Golgi trafficking such as Sec12 and Sec20, were identified. In *Candida albicans*, *O*-mannosylation positively affects the stability of the essential v-SNARE protein Sec20 that functions in retrograde transport from the Golgi to the ER (52), showing that *O*-Man glycans of ER proteins can directly affect protein performance. Furthermore, remarkable are the observed *O*-Man glycans in the ER-luminal PDIs and PDI-like proteins – Pdi1, Eug1, Mpd1, and Eps1, as well as the thiol oxidase Ero1 (Fig. 4). Oxidative protein folding in the ER is an essential function of eukaryotic cells. The yeast Ero1 oxidase transfers oxidizing equivalents to PDI, which transfers them to newly synthesized polypeptides leading to the formation or rearrangement of protein disulfide bonds (reviewed in (53)). Both, Ero1 and Pdi1 have been previously identified as interactors of PMTs (51). Ero1 contains *O*-Man glycans mainly in the essential *C*-terminal region that tethers the protein to ER membrane components (supplemental Fig. S3; (54)). In all yeast PDIs the *O*-Man glycans are in the proximity of the catalytic CysXXCys motifs, where also *N*-linked glycans are present ((53), Fig. 4*A*). It remains a challenging task to unravel the functions of these glycans for ER and Golgi homeostasis in the future.

##### Sequence Motifs for O-Mannosylation?

In yeast, the PMT family is highly redundant (Pmt1–6) and falls into three subfamilies (PMT1, PMT2, PMT4) (reviewed in (11)). Pmt1/Pmt2 and Pmt4 act on distinct proteins, but despite the fact that Pmt4 preferentially modifies membrane-anchored proteins, parameters that define substrate recognition have not been identified yet (18, 37). The *O*-Man glycoproteome revealed various but indistinct characteristics of *O*-Man sites, which might be attributed, at least in part, to the different substrate and/or glycosylation site specificities of the yeast PMT family members. As anticipated, the majority of *O*-Man sites are situated in protein regions of about 20–50% Ser/Thr-content, which are hydrophilic in nature and often present intrinsically disordered domains (Figs. 3, 5). We further observed that *O*-mannosylation is favored in regions with higher probability to form β-strands (Fig. 5). In accordance with our data, a recent study investigating the conformational properties of different *O*-linked sugars using force field molecular dynamics simulation showed the conformational stabilization of β-strands and polyproline type II conformations over α-helices via intramolecular hydrogen bonds and bridging water molecules (55).

We found that like in humans, *O*-mannosylation of Thr is favored over Ser (Fig. 5; (21, 56)). In yeast, apart from hydroxyl-amino acids, Ala and Val residues are preferred in the proximity of the *O*-Man sites, whereas Asp and Leu residues are less favored (Fig. 5). Our findings are in good agreement with previous *in vitro* studies addressing PMT acceptor sites, using synthetic penta-peptides (19, 47). In mammals, Pro residues support the nearby addition of *O*-GalNAc residues (57). Although previous studies on synthetic penta-peptides as well as the here identified ProThr-motif on PDI suggest similar for yeast *O*-mannosylation ((47); Fig. 4*C*), a significant overall enrichment of Pro in the proximity of *O*-Man glycosites was not observed (Fig. 5). Having established a shotgun strategy to probe the *O*-Man glycoproteome we can now move forward with studying PMT isoform specificities similar to what has recently been developed for *O*-GalNAc using quantitative differential *O*-glycoproteomics (58).

##### Interplay with N-Glycosylation

Our study revealed that at the sequon sequence Asn-X-Ser/Thr, *N*- is favored over *O*-glycosylation and that in the immediate vicinity of *N*-glycosylation acceptor sites *O*-mannosylation is also less prevalent (Figs. 5 and 6). A hallmark of *N*-glycosylation is the requirement of a Ser or Thr residue at the +2 position of the acceptor site, because the corresponding hydroxyl group is important for the positioning of the peptide substrate to the active site of OST (59). Hence, *O*-mannosylation of the acceptor sequon would render *N*-glycosylation virtually impossible. In agreement, we only found few reported *N*-glycosylation sequons to be *O*-mannosylated. Also *in vitro N*-glycosylation of the model substrate TSSQATSS clearly predominated (Fig. 6). However, when this sequence is placed *C*-terminal of an extended Ser/Thr-rich region, as naturally occurring in the cell wall protein Ccw5, *O*-mannosylation is favored (8, 13). Overall, these data show that the mutual influence between the two glycosylation types at *N*-glycan acceptor sites depends very much on the surrounding protein context. The interplay of *O*- and *N*-glycosylation is almost certainly a considerable factor determining micro- (*i.e.* local structure/conformation of glycans) and macro- (*i.e.* glycan site usage) heterogeneity of glycoproteins in yeast and mammals, and will be an interesting subject toward deciphering the undoubtedly immense role of protein glycosylation.

##### Yeast versus Mammalian O-glycosylation

In fungi, *O*-mannosylation is the only type of *O*-glycosylation, whereas in higher eukaryotes *O*-mannosylation is so far mainly restricted to specific cell surface and extracellular matrix proteins such as α-dystroglycan, KIAA1549, members of the cadherin and plexin superfamilies and lecticans (21, 60). Although cell surface proteins vary substantially between yeast and mammals, basic characteristics of *O*-mannosylation are highly similar ((21); see above). Besides cell surface decoration and hence cell wall organization or cell adhesion, respectively, our study now provides clear indications for further conserved, but so far unrecognized functions of *O*-mannosylation, as in the case of PDI (Fig. 4; see above).

In contrast to yeast, however, mucin-type *O*-GalNAc glycosylation became the most common covalent modification of Ser and Thr residues in mammals. Analysis of orthologs proteins from yeast and human revealed a set of common substrates, and glycodomain classification and sequence alignments showed a partial overlap of glycosites or domains. Yeast *O*-Man and human *O*-GalNAc glycosylation share the same prevalence for acceptor sites outside of protein folding domains. However, *O*-mannosylation is less prevalent in linker or stem regions (Fig. 3), but more frequently found in the *C*- and *N*-terminal regions (data not shown). Positions of glycosylation suggest direct conserved biological functions for selected proteins. As an example, the human aspartyl proteases cathepsin D (CTSD) and yeast proteinase A (Pep4) are putative orthologs (supplemental Fig. S3). Their alignment shows a high degree of sequence conservation in their catalytic domains. For both enzymes, there is a glycosylation site in the activation peptide, which is the linker between the propeptide and the catalytic domain. At the carboxy-terminal of this peptide, both orthologous proteins are cleaved to produce the mature protein, and both sets of glycosylation sites exhibit proximity to cleavage sites suggesting protection from propeptide removal (61). Similar mechanisms were recently described for the aspartic endopeptidase Yps1, where *O*-mannosylation regulates its shedding activity of GPI-anchored proteins (62).

In summary, our study provides a deep insight into the *O*-Man glycoproteome of yeast and shows that *O*-Man glycosylation is found widely not only on cell wall proteins but also ER and Golgi proteins. *O*-mannosylation of some orthologous proteins is highly conserved between yeast and mammals. However, in most mammalian proteins *O*-Man glycans appear to have been replaced by *O*-GalNAc glycosylation, which may conserve the general function of the protein-linked carbohydrate, but in addition, may introduce new beneficial physicochemical properties to the protein (55).

## Supplementary Material

Supplemental Data

## Footnotes

^1^ The abbreviations used are:

*O*-Man: *O*-mannose, *O*-mannosyl
*O*-GalNAc: *O*-linked N-acetylgalactosamine
ETD: electron-transfer dissociation
ER: endoplasmic reticulum
HCD: higher-energy collision-induced dissociation
LWAC: lectin weak affinity chromatography
OST: oligosaccharyl transferase
PMT: POMT, protein *O*-mannosyltransferase
WT: wild-type
IEF: isoelectric focusing
HPEAC-PAD: high-performance anion-exchange liquid chromatography with pulsed amperometric detection.
