# Supplementary material for: Mapping the O-Mannose Glycoproteome in Saccharomyces cerevisiae
Source: Mol Cell Proteomics. 2016 Jan 13;15(4):1323–37. doi: 10.1074/mcp.M115.057505 (PMC4824858; doi:10.1074/mcp.M115.057505)
Supplement: Supplemental Data [file supp_15_4_1323__index.html]

Mapping the O-mannose glycoproteome in Saccharomyces cerevisiae — Mapping the O-mannose glycoproteome in Saccharomyces cerevisiae — Mapping the O-Mannose Glycoproteome in Saccharomyces cerevisiae — The O-Mannose Glycoproteome in Baker's Yeast — Supplemental Data 

# Mapping the *O*-Mannose Glycoproteome in *Saccharomyces cerevisiae*

## Supplemental Data

- Supplemental Data (.pdf, 11.4 MB) - Supplemental Experimental Procedures, Supplemental Tables S1-S6 and Supplemental Figures S1-S3
- Supplemental Table S2 (.xlsx, 743 KB) - Interactive listings of all identified O-Man glycoproteins including mapped *O*-Man glycosites from total cell extracts of *S. cerevisiae*.
- Supplemental Table S3 (.xlsx, 511 KB) - List of all identified *O*-Man glycopeptides and mapped *O*-Man glycoproteins from total cell extracts of *S. cerevisiae*.
- Supplemental Table S4 (.xlsx, 38 KB) - List of all identified *O*-Man glycopeptides and mapped *O*-Man glycoproteins from isolated cell walls of *S. cerevisiae*.
- Supplemental Table S5 (.xlsx, 4.4 MB) - Calculation of Ser/Thr content by sliding window analysis.
- Supplemental Table S6 (.xlsx, 46 KB) - *In silico* digest of cell wall proteins.
- Supplemental Figure S3 (.pdf, 800 KB) - Alignments of human orthologues *O*-glycoproteins.
