# Supplementary material for: Mapping the O-Mannose Glycoproteome in Saccharomyces cerevisiae
Source: Mol Cell Proteomics. 2016 Jan 13;15(4):1323–37. doi: 10.1074/mcp.M115.057505 (PMC4824858; doi:10.1074/mcp.M115.057505)
Supplement: Supplemental Data [file 10.1074_M115.057505_mcp.M115.057505-1.pdf]

Mapping the *O*-mannose glycoproteome in *Saccharomyces cerevisiae*

Patrick Neubert[1*], Adnan Halim[2*], Martin Zauser[1], Andreas Essig[3], Hiren J. Joshi[2], Ewa Zatorska[1], Ida Signe Bohse Larsen[2], Martin Loibl[1], Joan Castells-Ballester[1], Markus Aebi[3], Henrik Clausen[2], Sabine Strahl[1§]

Supplemental Experimental Procedures

Supplemental Tables S1-S6

Supplemental Figures S1-S4

References

SUPPLEMENTAL EXPERIMENTAL PROCEDURES

Yeast strains and plasmids — *S. cerevisiae* strains used in this study are listed in Supplemental Table S1**.** Cells were grown in YPD (1% yeast extract, 2% peptone, 2% glucose) under standard conditions. Transformations were transformed according to Gietz *et al.* (1). All yeast transformants were validated by PCR analysis and plasmids by DNA sequencing.

Plasmid pLF15B (TSTQATSS): A synthetic gene encoding the Suc2 signal peptide (Met1 to Val31) (55) fused to a truncated version of Ccw5 (Ser119 to Cys227) with all Ser and Thr residues strating from Ser119 replaced for Ala was made by GeneArt® (Life Technologies, Germany). Using PCR (oligos 2404 and 2405), the BamHI and PstI restriction site was introduced at the 5'- and 3'-end of the synthetic gene, respectively. The obtained PCR fragment was cloned via BamHI and PstI into plasmid pML60 (6). The resulting plasmid pLF3, encoding for the synthetic Ccw5 protein (Ala119 to Cys227) C-terminally fused to FLAG tag, was digested with SphI and NotI and ligated with annealed oligo pair 2396 and 2397 (encoding for Ccw5 from Lys110 to Ser118). The resulting plasmid pLF15 (encoding for synthetic Ccw5 from Lys110 to Cys227) was digested with BamHI and SphI and ligated with the annealed oligo pair 1985 and 1986. The obtained plasmid pLF15B encodes for synthetic Ccw5 (Lys110 to Cys227) fused at its N-terminus to the Suc2 signal peptide with Met21 exchange for Val, and Asn23 for Gln.

Plasmids pLF25, pLF30-32 and pLF34: Annealed oligo pairs were cloned into plasmid pLF15B digested with SphI and NotI. For pLF25 (TSTNATSS) oligos 2126 and 2127, for pLF30 (NATSTQATSS) oligos 2128 and 2129, for pLF31 (TSTQATSSNAT) oligos 2130 and 2131, for pLF32 (NATggpglTSTQATSS) oligos 2132 and 2133, and for pLF34 (TSTQATSSggpglNAT) oligos 2136 and 2137 were used. Oligonucleotide sequences are available upon request.

Cell free microsomal translation/translocation assay — Synthesis of 5′-capped mRNA was performed using SP6 RNA polymerase (gift from B. Dobberstein) from linearized plasmid templates according to High *et al.* (2). Preparation of yeast microsomes (strain SEY6210), translation (from 80 ng of mRNA) and translocation assays ([35S]methionine/cysteine was omitted) were performed as described recently (3). The reactions were incubated for 1 h at 30 °C. Post-translational protease protection assays were performed as described (4). When necessary, microsomes were purified using a sucrose cushion (5). Proteins were separated on 15% polyacrylamide gels and detected by Western blot using the anti-FLAG (M2, Sigma-Aldrich, Germany) monoclonal antibodies at a dilution of 1:10,000.

For Endo H treatment microsomes (24 µg) were purified, resuspended in denaturing buffer (NEB, Frankfurt/Main, Germany) and heated at 65 °C for 15 min. Thereafter, 250 U of Endo H (NEB, Frankfurt/Main, Germany) were added and samples incubated at 37°C for 1 h in the presence of 5 mM PMSF.

For lectin enrichment microsomes (24 µg) were purified and resuspended in 0.5% (w/v) SDS, q. Proteins were solubilized at 99 °C for 5 min. Binding to Con A sepharose beads (GE Healthcare, Germany) was performed over night at 4°C in 20 mM ammonium bicarbonate buffer containing 500 mM NaCl, 1 mM $MnCl_2$ and 1 mM $CaCl_2$. Alternatively, proteins were immunoprecipitated using anti-FLAG magnetic beads (Sigma-Aldrich, Germany).

For LC-MS/MS analyses, FLAG-tagged proteins were immunoprecipitated from microsomal reactions and isolated by SDS-PAGE. Peptides were created by in-gel digestion with trypsin and analyzed according to Winterhalter *et al.* (6).

SUPPLEMENTAL TABLE S1:

List of *Saccharomyces cerevisiae* strains used in this study.

Strain	Genotype	Reference
BY4741 (WT)	*MATa met15Δ0 his3*Δ1 *leu2Δ0 ura3Δ0*	EUROSCARF
*kre2*Δ	BY4741 except *kre2Δ::KanMX4*	EUROSCARF
*ktr1*Δ	BY4741 except *ktr1Δ::KanMX4*	EUROSCARF
*ktr3*Δ	BY4741 except *ktr3Δ::KanMX4*	EUROSCARF
kre2Δktr1Δktr3Δ (KTRΔ)	BY4741 except *kre2Δ::His3-GFP ktr1::SAT ktr3::KanMX4*	(A gift of Horward Bussey)
SS328	*MATa ade2-101 his3Δ200 ura3-52 lys2-801*	te Heesen *et al.* (1993) (7)
SEY6210	*MATα leu2-3,112 ura3-52 his3-Δ200 trp1-Δ901 ade2-101 suc2-Δ9*	Robinson *et al.* (1988) (8)

Supplemental Table S2.

Interactive listings of all identified O-Man glycoproteins including mapped O-Man glycosites from total cell extracts of S. cerevisiae. The Excel file contains all *S. cerevisiae* proteins recorded by commonly used identifiers (UniprotID, SGD-ID, systematic name and standard name). Further, all information about subcellular localization is listed that was gathered from manual curation in the SGD database and from manually selected, high-confidence scored cellular component GO annotations in the Compartments DB. Additional information about various classifications and predictions is included (i.e., GPI-anchor, signal peptide predictions, essentiality). Proteins from the yeast secretome are highlighted, and information about SRP-dependence of ER translocation is provided (extracted from Ast *et al.* (9)). Experimentally described *N*-linked glycoproteins and the corresponding N-glycosylation sites are shown (extracted from Zielinska *et al.* (10)). *O*-Man glycoproteins predicted by application of the NetOGlyc algorithm the yeast glycoproteome are included (extracted from González *et al.* (11)). Finally, *O*-Man glycoproteins identified in this study are highlighted and information about numbers and positions of *O*-Man sites are provided. Number and sites of *O*-mannosylation are accounted both combined and separately. Unambiguous sites were assigned by ETD-MS/MS and ambiguous sites were mapped from HCD-MS/MS. *O*-Man glycoproteins without site information were identified solely by the hexose substraction routine (see Experimental Procedures for methodical details).

[Separate Excel file – Supplemental_Table_S2.xlsx]

Supplemental Table S3:

Listing of all identified O-Man glycopeptides and mapped O-Man glycoproteins from total cell extracts of S. cerevisiae. The Excel file contains all *O*-Man glycopeptide sequences identified by LC-MS/MS from total cell lysates. Small letters indicate modification events: carbamidomethylation on Cys, oxidation of Met, deamidation of Asn and hexosylation of Ser and Thr. In addition, protein assignment including the full name and the commonly used short form, UniProt accession, number of modification per position (HCD, ETD) or peptide (neutral loss of hexoses, marked as "CID") are shown. MS analysis was performed on Con A enriched glycopeptide fractions prepared from WT and *KTR*Δ

cells. In total, data were collected from eight independent preparations (indicated in column "biological replicate #"). Total cell lysates from *KTR*Δ were digested by trypsin in triplicates, and digested by GluC and chymotrypsin in duplicates. Additionally, data were collected from WT cell extracts digested by trypsin once (see Experimental Procedures for methodical details).

[Separate excel file – Supplemental_Table_S3.xlsx]

SUPPLEMENTAL TABLE S4.

Listing of all identified O-Man glycopeptides and mapped O-Man glycoproteins from isolated cell walls of S. cerevisiae. The Excel file contains all *O*-Man glycopeptide sequences identified by LC-MS/MS from isolated cell wall proteins. For the LysN digest, HCD-MS/MS was performed with the same extract as prepared for ETD. For the LysC digest, the ETD and HCD measurement was performed with two independently prepared extracts. ETD was solely used for the extract digested with AspN (see Experimental Procedures for methodological details). In addition protein assignment including UniProt accession and the commonly used short name, the preparation the results originate from, HCD and ETD data are shown. Peptides containing a PIR repeat which could not be assigned to a specific protein are included. Oxidation of methionine is indicated by an M next to the number of mannoses (# Man) attached to the peptide.

[Separate Excel file – Supplemental_Table_S4.xlsx]

SUPPLEMENTAL TABLE S5.

Calculation of Ser/Thr content by sliding window analysis. The Excel file includes the analysis of the Ser/Thr-content of 333 proteins that are translocated into the ER in an SRP-independent manner (according to Ast *et al.*(9)). Sliding window analysis was performed using the in-house prepared "R" script. A default window size of 21 amino acids (highlighted in green) was chosen. Colors in the value table highlight a Ser/Thr-content >30% (red), >40% (yellow) and >50% (green).

[Separate Excel file – Supplemental_Table_S5.xlsx]

SUPPLEMENTAL TABLE S6.

In silico digest of cell wall proteins. The Excel file shows all potential MS-applicable peptides of a typical length of 6 to 25 amino acids that could be obtained from the cell wall proteins shown in Figure 3, by digestion with the endoproteinases trypsin, AspN, LysN and LysC. *In silico* digestion was performed using the ExPASy tool "Peptide Cutter". The proven *O*-Man glycopeptides are highlighted in green.

[Separate Excel file – Supplemental_Table_S6.xlsx]

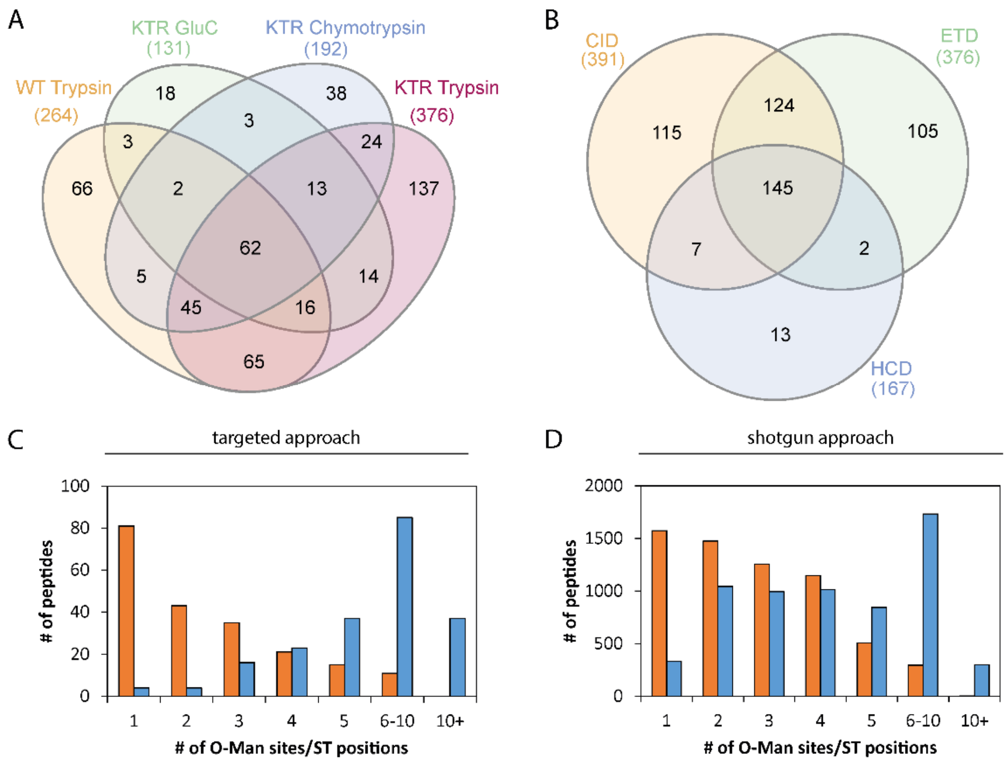

SUPPLEMENTAL FIGURE S1:

(A) Venn diagram illustrating the coverage of *O*-Man glycoproteins in different datasets utilizing different proteases and strains. Data were collected from 8 independent datasets, KTRΔ was digested with trypsin in triplicates and with GluC and Chymotrypsin in duplicates each. WT yeast was digested with trypsin once. **(B)** Venn diagram illustrating the coverage of *O*-Man proteins identified from different activation types (HCD and ETD) and the hexose mass substraction routine (marked as “CID”) **(C, D)** Bar plots analyzing the number of *O*-Man sites (orange) and the number of Ser/Thr positions (blue) that could be identified per peptide comparing **(C)** the cell wall targeting approach utilizing α-mannosidase trimming and **(D)** the shotgun approach from glycan elongation impaired KTRΔ cells. **(D)** Assuming every Ser or Thr residue could be a potential *O*-Man site, about 32 % of the glycopeptides were fully glycosylated. In average, 2.7 *O*-Man sites per peptide were detected.

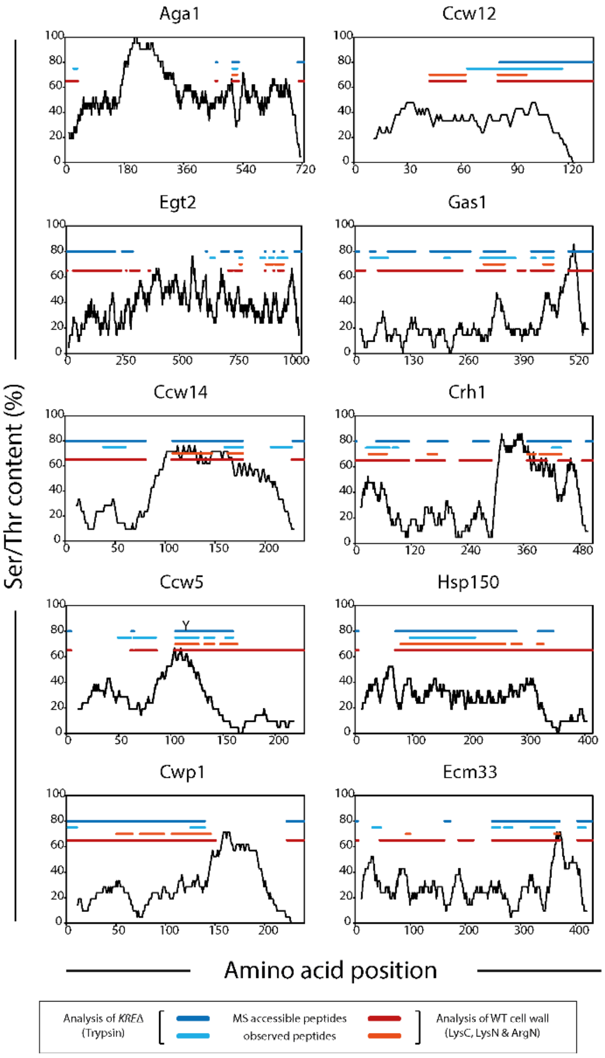

Supplemental Figure S2:

Peptide coverage of selected cell wall *O*-Man glycoproteins. Ten examples of *O*-Man glycoproteins are represented by sliding window blots calculating the Ser/Thr content in percent. MS-applicable and identified (glyco-)peptides are depicted as colored lines. Glycopeptides from independent experiments utilizing either trypsin digestion with subsequent Con A enrichment of *KTR*Δ total cell lysates (light-blue) or a combination of LysC, LysN and ArgN digestion of α-mannosidase treated enriched cell wall fractions (light-red), are shown. Considering the presence of cleavage sites and peptides of a typical length of 6 to 25 amino acids, potential MS-applicable protein regions are depicted in the corresponding darker colors. Both approaches generate highly comparable results limited only by the presence of applicable cleavage sites. Results for calculating the Ser/Thr content can be found in Supplemental Table S5. Information from the in silico digest were summarized in Supplemental Table S6.

SUPPLEMENTAL FIGURE S3.

Alignments of human orthologues *O*-glycoproteins. A selection of human orthologs carrying *O*-GalNAc modifications are presented and compared to here identified yeast *O*-Man glycoproteins. Depicted are the positions of modifications relative to the aligned protein sequences. Relative degree of conservation is indicated by a greyscale bar code.

[Separate pdf file – Supplemental_Figure_S3.pdf]

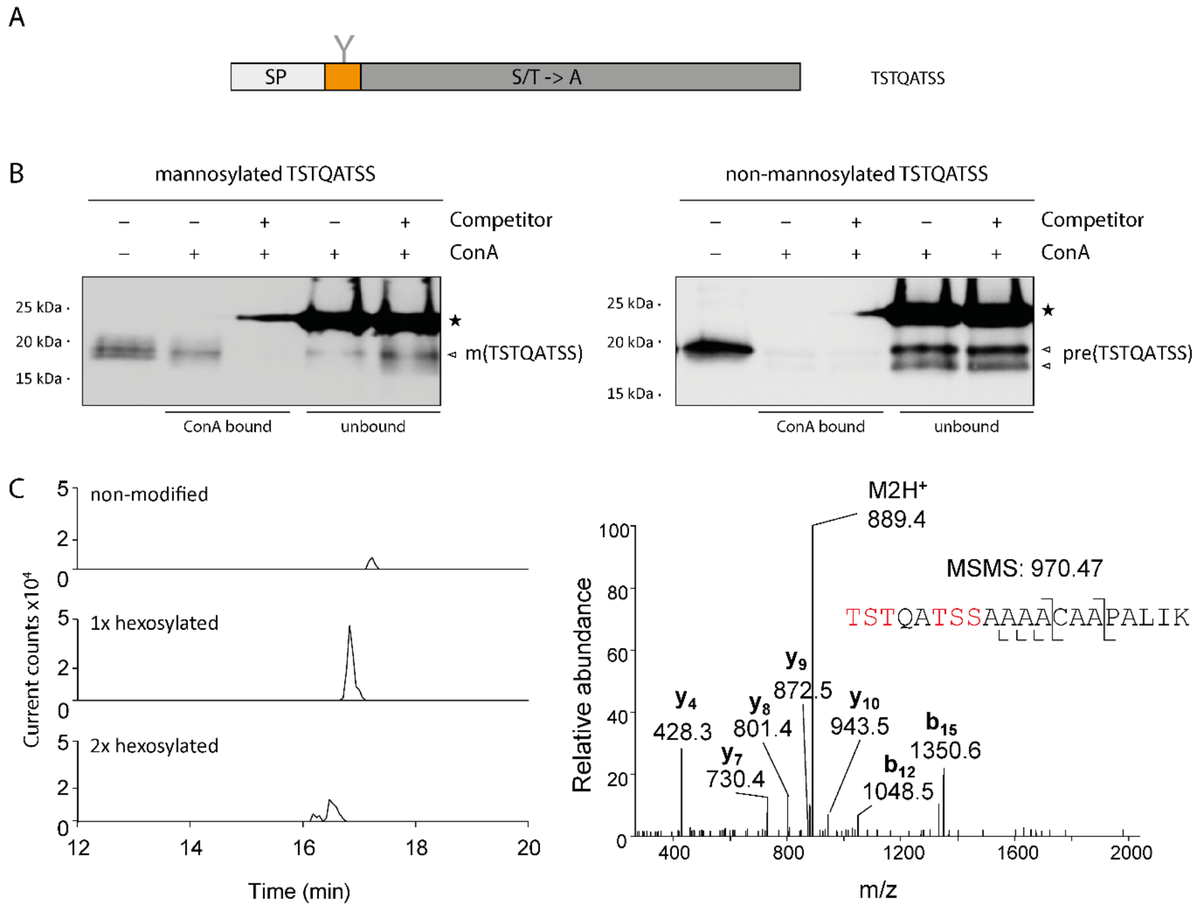

Supplemental Figure S4.

Model acceptor protein TSTQATSS is an *O*-Man substrate. (A) Schematic representation of acceptor model TST(Q/N)ATSS. The sequence originates from the cell wall protein Ccw5 (REF: Loibl) and was isolated from its Ser/Thr context by alanine shift of remaining potential O-Man sites. In orange, the O-Man acceptor sequence TST(Q/N)ATSS is highlighted. **(B)** Con A pull down of protein TSTQATSS (open triangle), after (mannosylated) and before (non-mannosylated) translocation into yeast microsomes. Solubilized microsomal proteins were affinity purified using lectin Con A in the absence or presence of the lectin binding competitor methyl-α-D-mannopyranoside (final concentration: 200 mM). Proteins from the unbound fraction were enriched via immunoprecipitation using anti-FLAG magnetic beads. Proteins were separated by SDS-PAGE and analyzed by Western blot using anti-FLAG antibody. Asterisks indicate the light chain of the FLAG antibody. **(C)** LC-MS/MS analysis of ConA-enriched TSTQATSS. Shown are the extracted ion chromatogram of non-hexosylated, one-time and two-times hexosylated TSTQATSS as well as the MS/MS spectra of m/z 970.47.

REFERENCES

1. Gietz, D., St Jean, A., Woods, R. A., and Schiestl, R. H. (1992) Improved method for high efficiency transformation of intact yeast cells. *Nucleic Acids Res* 20, 1425

2. High, S., Martoglio, B., Gorlich, D., Andersen, S. S., Ashford, A. J., Giner, A., Hartmann, E., Prehn, S., Rapoport, T. A., Dobberstein, B., and et al. (1993) Site-specific photocross-linking reveals that Sec61p and TRAM contact different regions of a membrane-inserted signal sequence. *J Biol Chem* 268, 26745-26751

3. Loibl, M., Wunderle, L., Hutzler, J., Schulz, B. L., Aebi, M., and Strahl, S. (2014) Protein O-mannosyltransferases associate with the translocon to modify translocating polypeptide chains. *J Biol Chem* 289, 8599-8611

4. Walter, P., and Blobel, G. (1983) Preparation of microsomal membranes for cotranslational protein translocation. *Methods Enzymol* 96, 84-93

5. Weihofen, A., Lemberg, M. K., Ploegh, H. L., Bogyo, M., and Martoglio, B. (2000) Release of signal peptide fragments into the cytosol requires cleavage in the transmembrane region by a protease activity that is specifically blocked by a novel cysteine protease inhibitor. *J Biol Chem* 275, 30951-30956

6. Winterhalter, P. R., Lommel, M., Ruppert, T., and Strahl, S. (2013) O-glycosylation of the non-canonical T-cadherin from rabbit skeletal muscle by single mannose residues. *FEBS Lett* 587, 3715-3721

7. te Heesen, S., Knauer, R., Lehle, L., and Aebi, M. (1993) Yeast Wbp1p and Swp1p form a protein complex essential for oligosaccharyl transferase activity. *EMBO J* 12, 279-284

8. Robinson, J. S., Klionsky, D. J., Banta, L. M., and Emr, S. D. (1988) Protein sorting in Saccharomyces cerevisiae: isolation of mutants defective in the delivery and processing of multiple vacuolar hydrolases. *Mol Cell Biol* 8, 4936-4948

9. Ast, T., Cohen, G., and Schuldiner, M. (2013) A network of cytosolic factors targets SRP-independent proteins to the endoplasmic reticulum. *Cell* 152, 1134-1145

10. Zielinska, D. F., Gnad, F., Schropp, K., Wisniewski, J. R., and Mann, M. (2012) Mapping N-Glycosylation Sites across Seven Evolutionarily Distant Species Reveals a Divergent Substrate Proteome Despite a Common Core Machinery. *Mol Cell* 46, 542-548

11. Gonzalez, M., Brito, N., and Celedonio, G. (2012) High abundance of Serine/Threonine-rich regions predicted to be hyper-O-glycosylated in the secretory proteins coded by eight fungal genomes. *BMC Microbiol* 12, 213
