# Supplementary material for: Mapping the O-Mannose Glycoproteome in Saccharomyces cerevisiae
Source: Mol Cell Proteomics. 2016 Jan 13;15(4):1323–37. doi: 10.1074/mcp.M115.057505 (PMC4824858; doi:10.1074/mcp.M115.057505)
Supplement: Supplemental Data [file 10.1074_M115.057505_mcp.M115.057505-7.pdf]

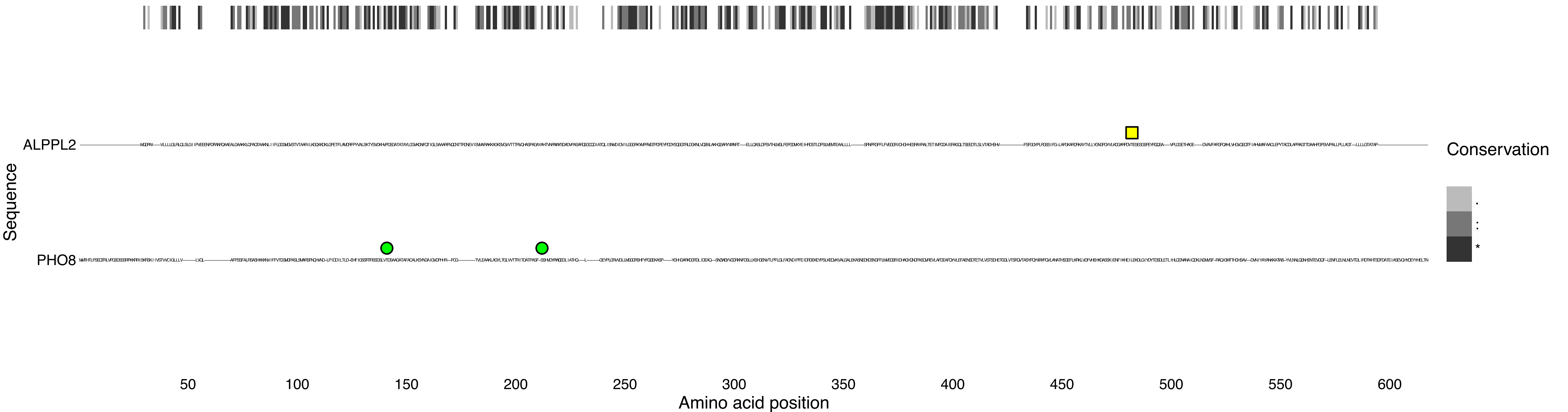
ALPPL2
PHO8
Sequence
Conservation
.
:
*
50
100
150
200
250
300
350
400
450
500
550
600
Amino acid position

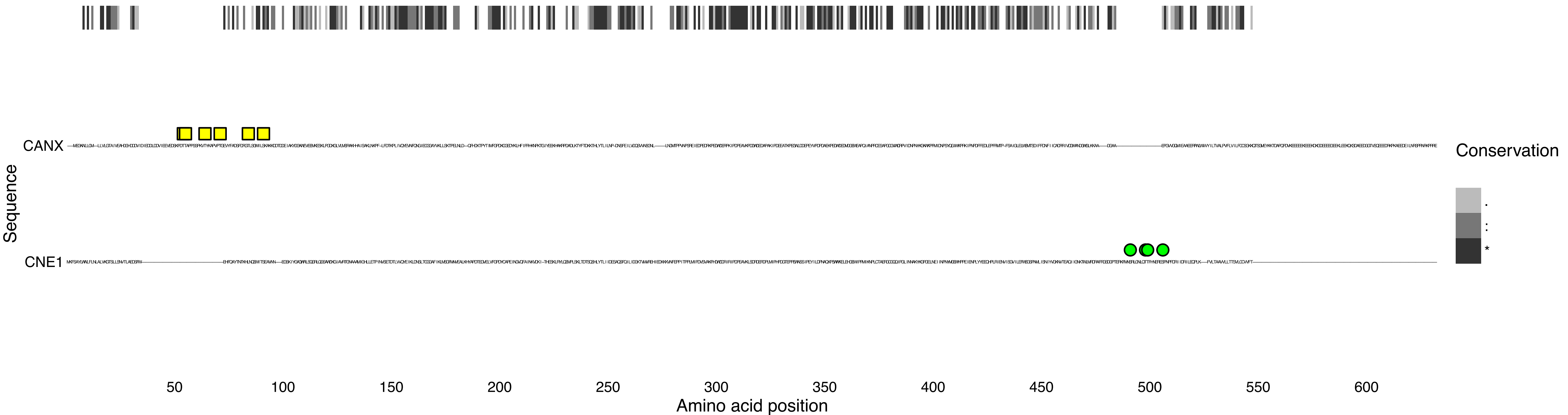
CANX
CNE1
Sequence
Conservation
.
:
*
50
100
150
200
250
300
350
400
450
500
550
600
Amino acid position

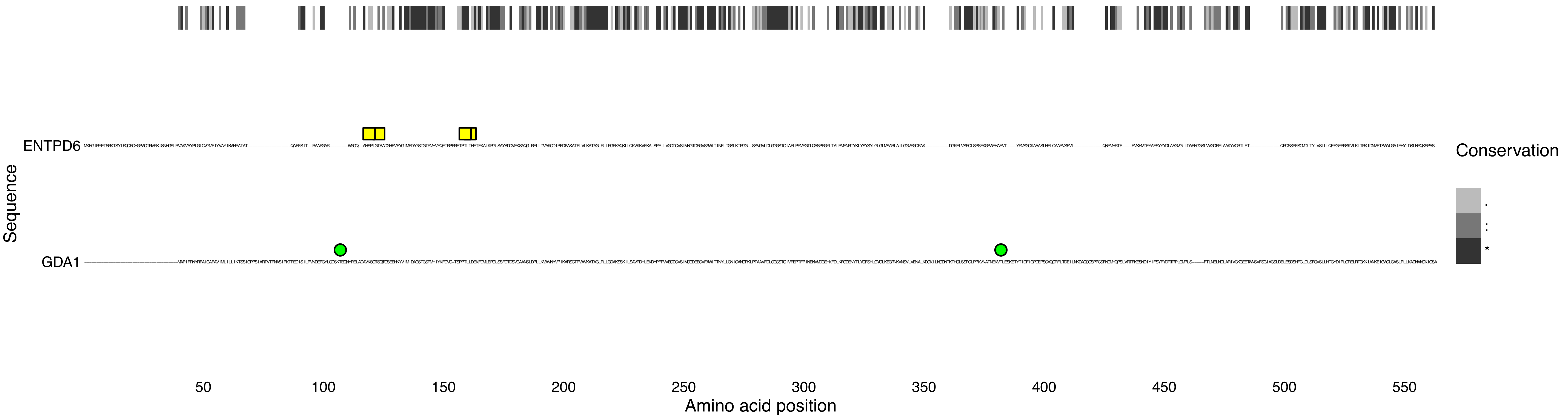
ENTPD6
GDA1
Sequence
Conservation
.
:
*
50
100
150
200
250
300
350
400
450
500
550
Amino acid position

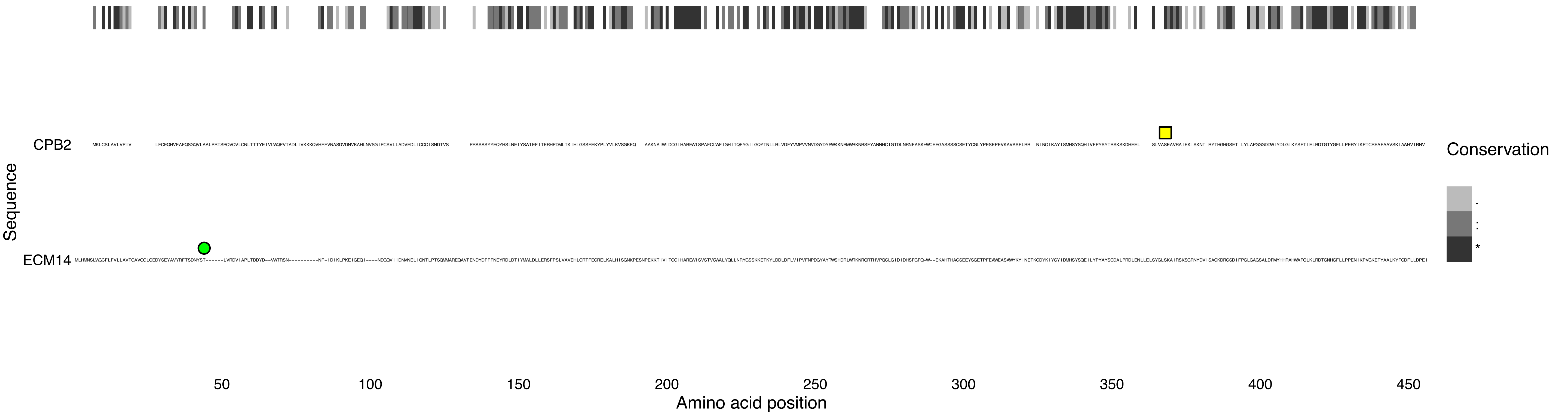
CPB2
ECM14
Sequence
Conservation
.
:
*
50
100
150
200
250
300
350
400
450
Amino acid position

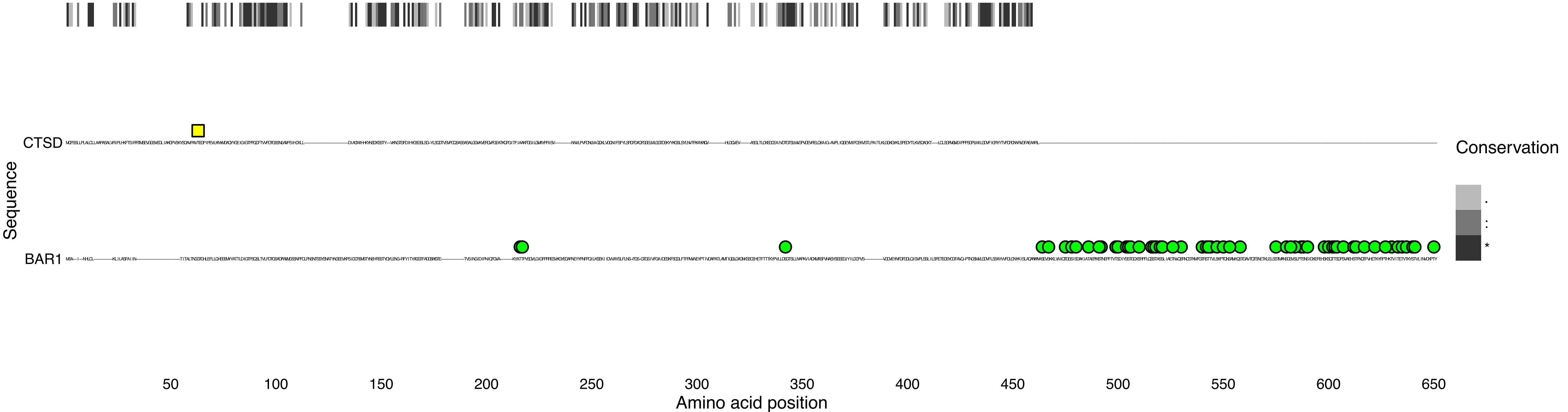
CTSD
BAR1
Sequence
Conservation
.
:
*
50
100
150
200
250
300
350
400
450
500
550
600
650
Amino acid position

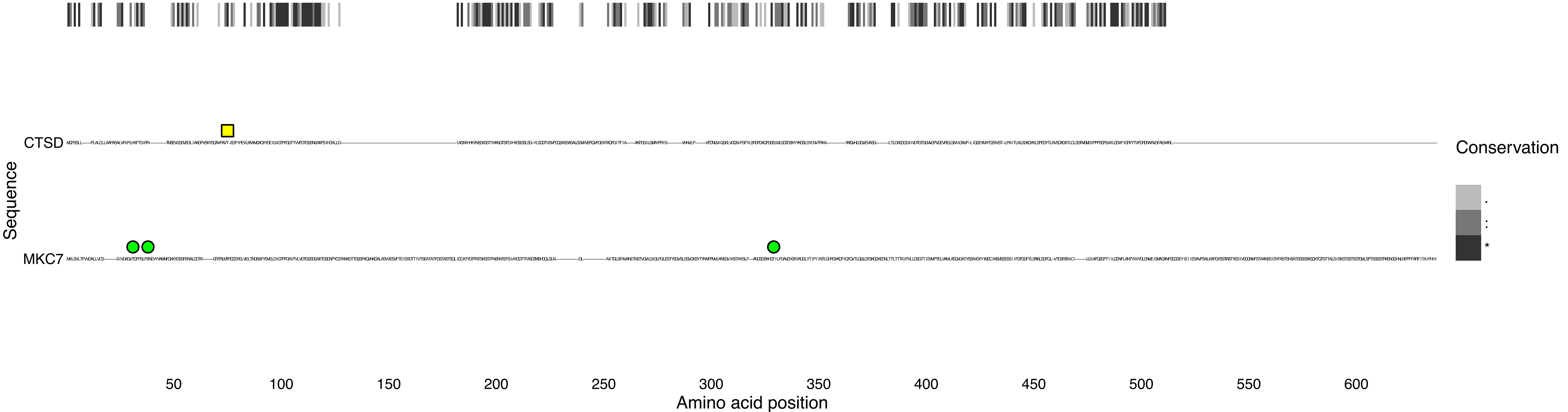

CTSD
MKC7
Sequence
Conservation
.
:
*
50
100
150
200
250
300
350
400
450
500
550
600
Amino acid position

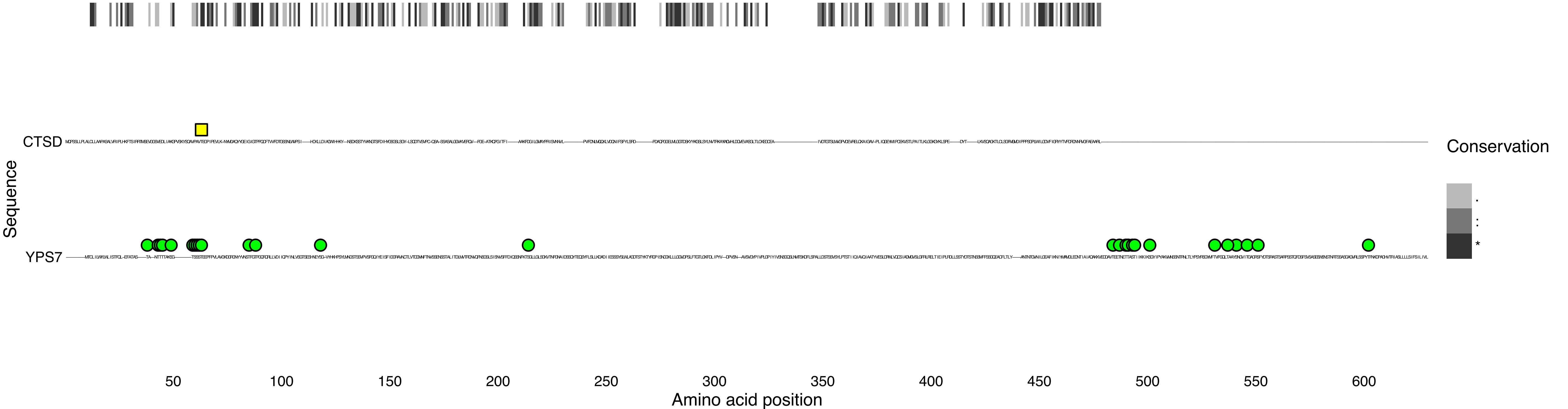
CTSD
YPS7
Sequence
Conservation
.
:
*
50
100
150
200
250
300
350
400
450
500
550
600
Amino acid position

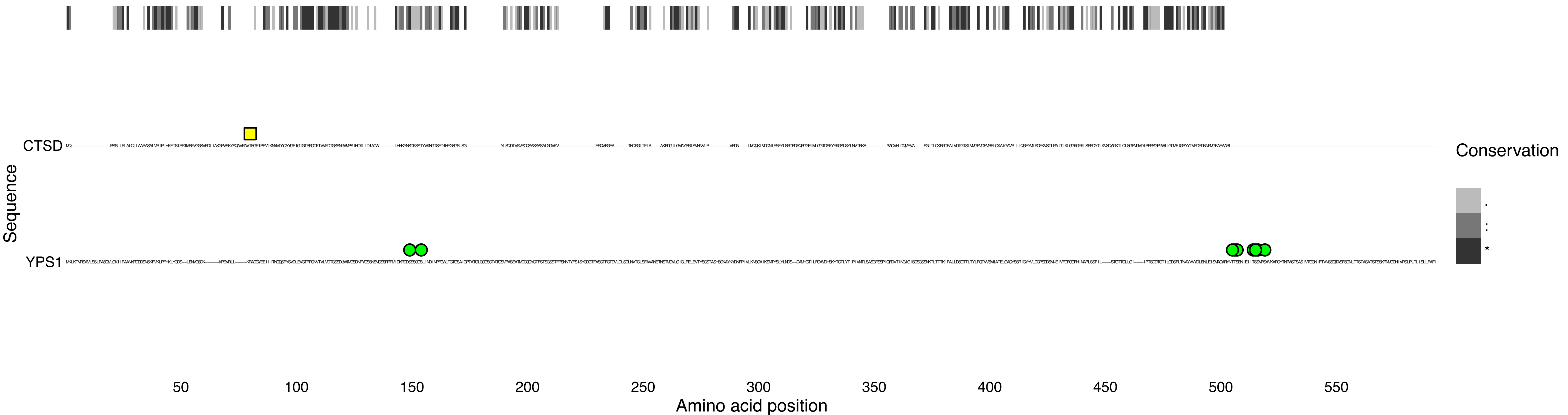

CTSD
YPS1
Sequence
Conservation
.
:
*
50
100
150
200
250
300
350
400
450
500
550
Amino acid position

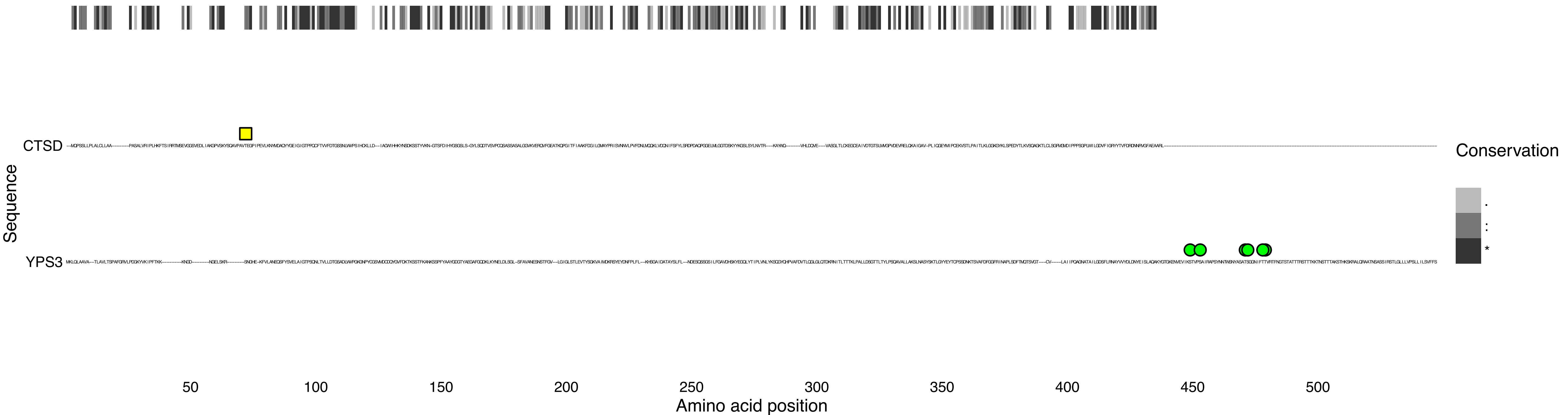
CTSD
YPS3
Sequence
Conservation
.
:
*
50
100
150
200
250
300
350
400
450
500
Amino acid position

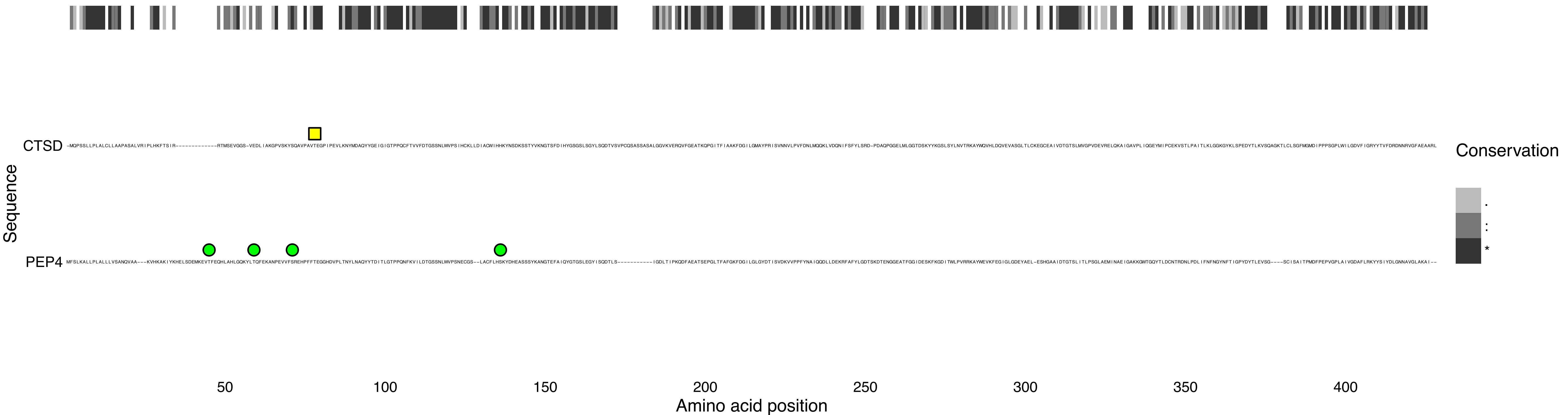
CTSD
PEP4
Sequence
Conservation
.
:
*
50
100
150
200
250
300
350
400
Amino acid position

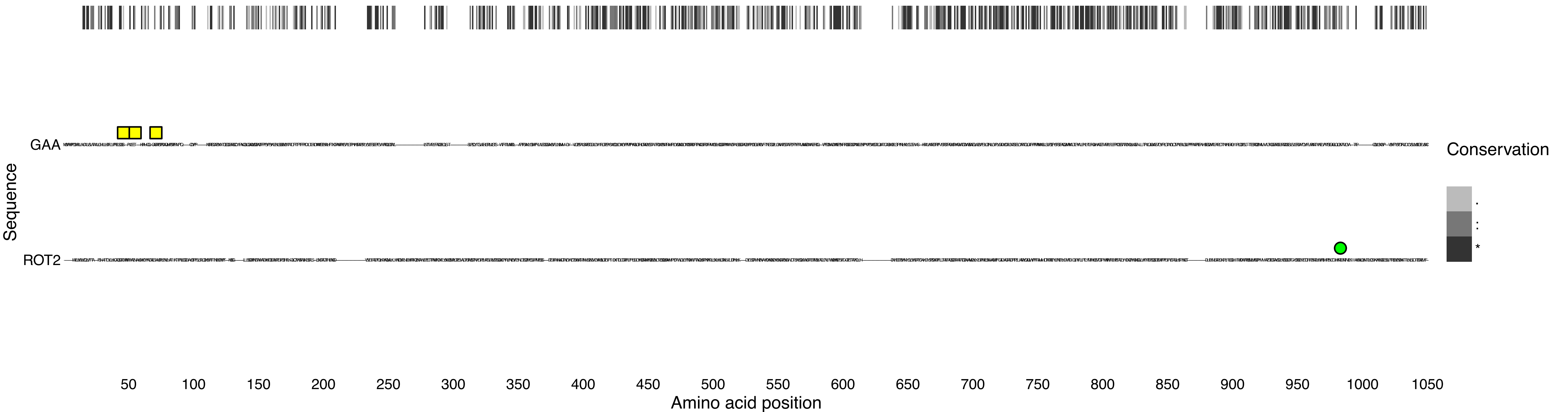
GAA
ROT2
Sequence
Conservation
.
:
*
50
100
150
200
250
300
350
400
450
500
550
600
650
700
750
800
850
900
950
1000
1050
Amino acid position

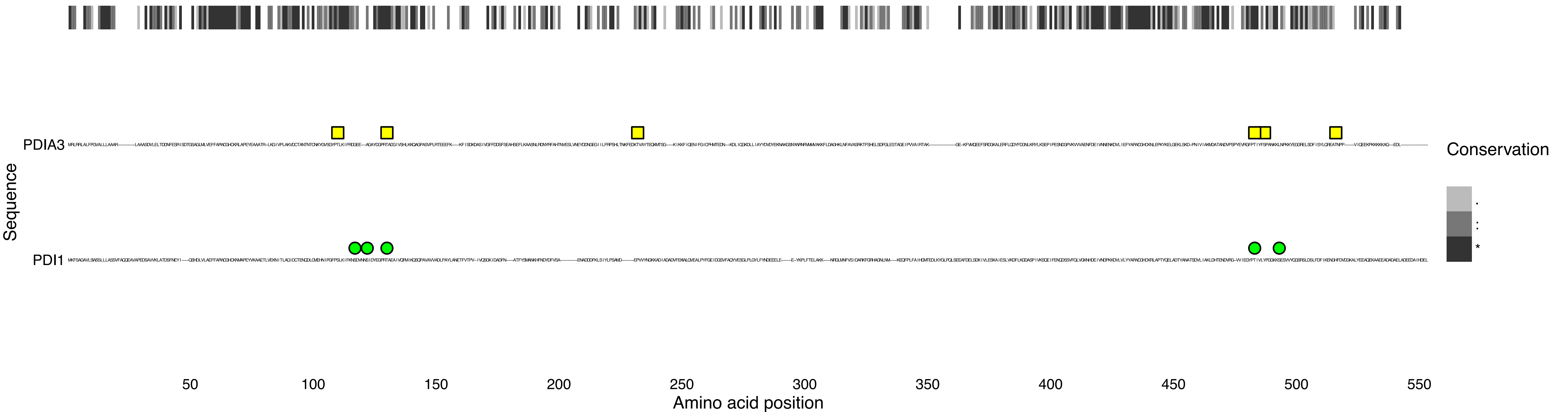
PDIA3
PDI1
Sequence
Conservation
.
:
*
50
100
150
200
250
300
350
400
450
500
550
Amino acid position

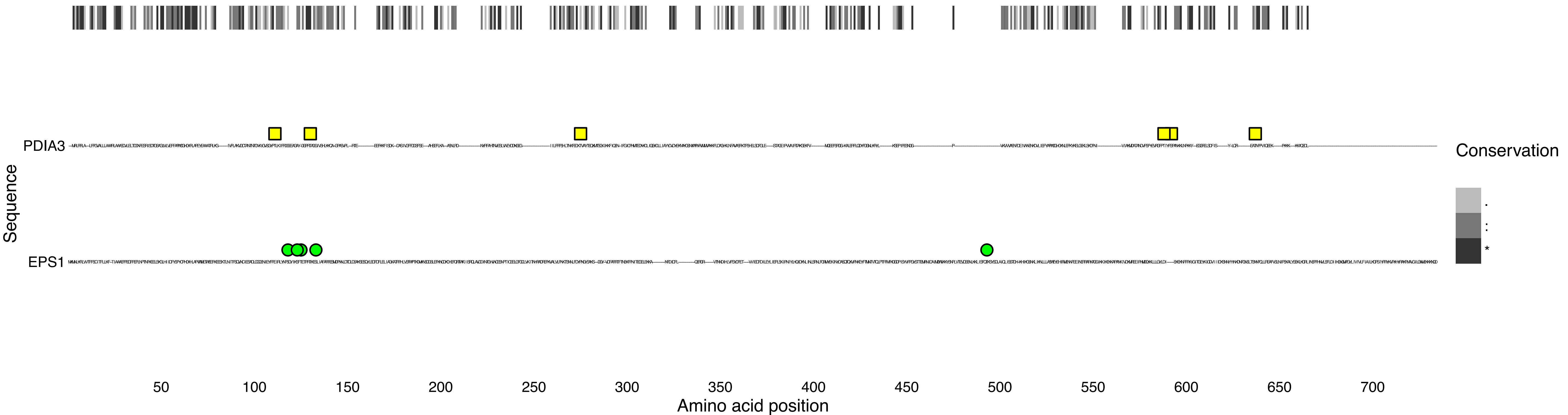

Conservation
.
:
*
PDIA3
EPS1
Sequence
50
100
150
200
250
300
350
400
450
500
550
600
650
700
Amino acid position

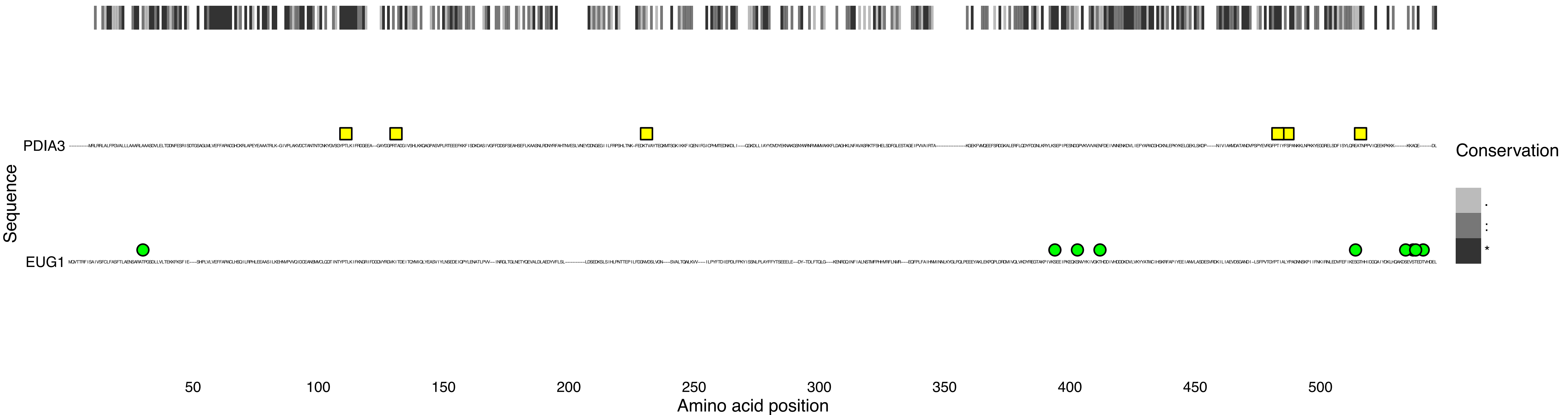

PDIA3
EUG1
Sequence
Conservation
.
:
*
50
100
150
200
250
300
350
400
450
500
Amino acid position

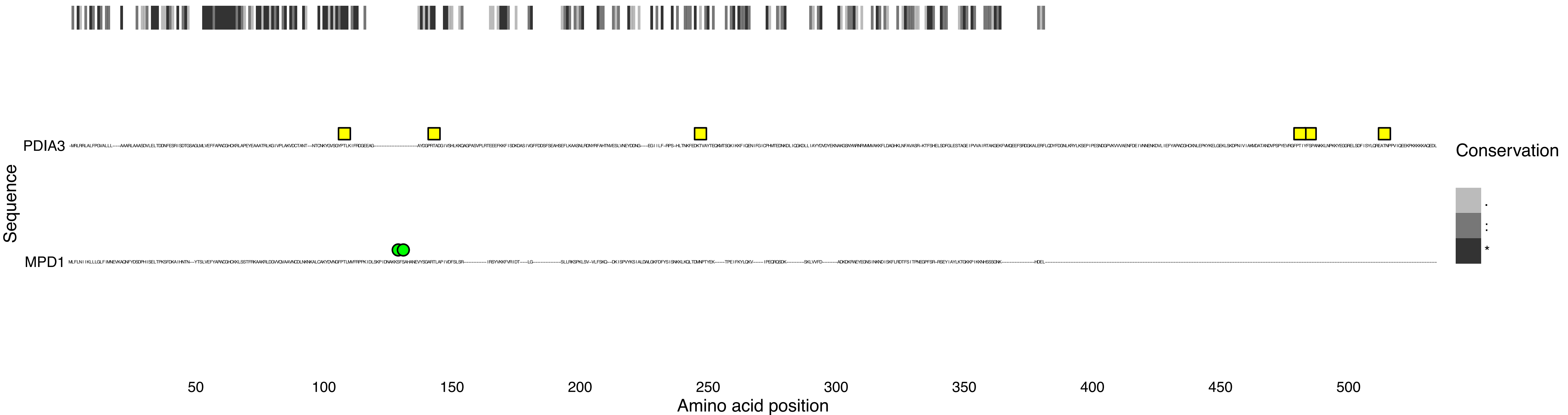
PDIA3
MPD1
Sequence
Conservation
.
:
*
50
100
150
200
250
300
350
400
450
500
Amino acid position

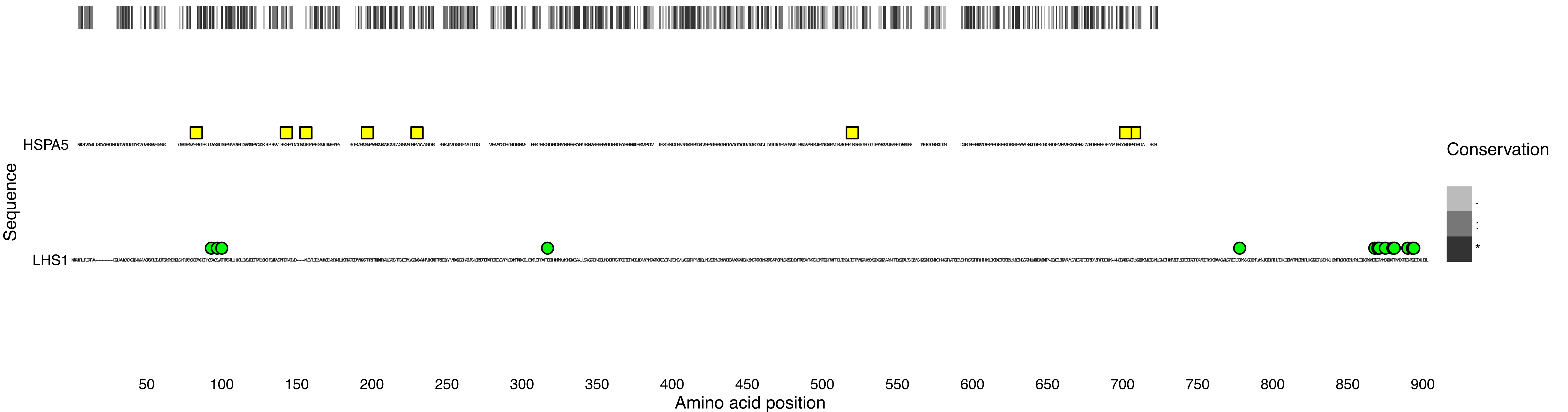
HSPA5
LHS1
Sequence
Conservation
.
:
*
50
100
150
200
250
300
350
400
450
500
550
600
650
700
750
800
850
900
Amino acid position

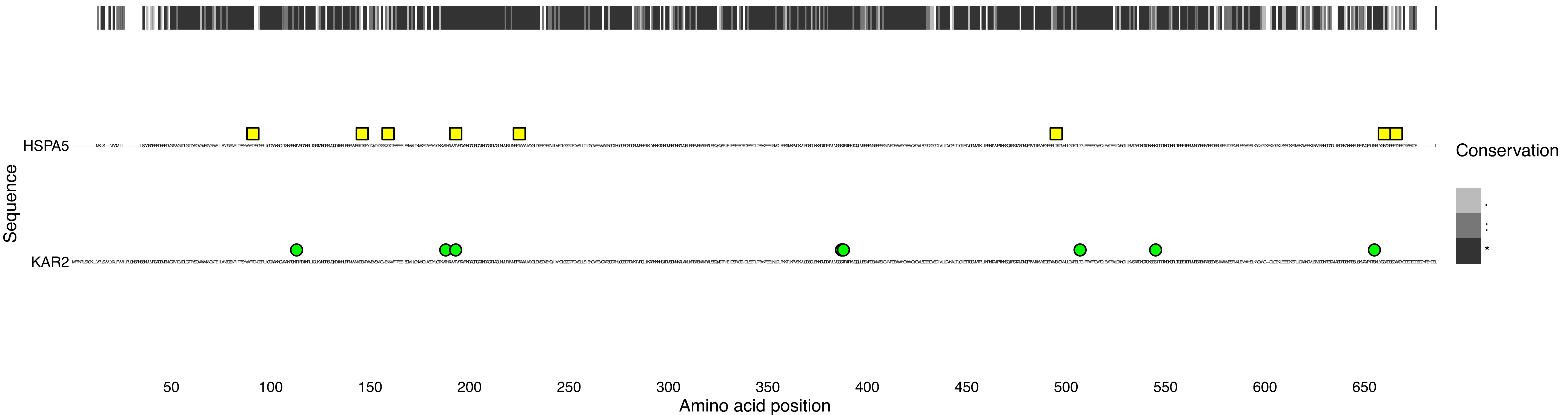
Conservation
.
:
*
HSPA5
KAR2
Sequence
50
100
150
200
250
300
350
400
450
500
550
600
650
Amino acid position

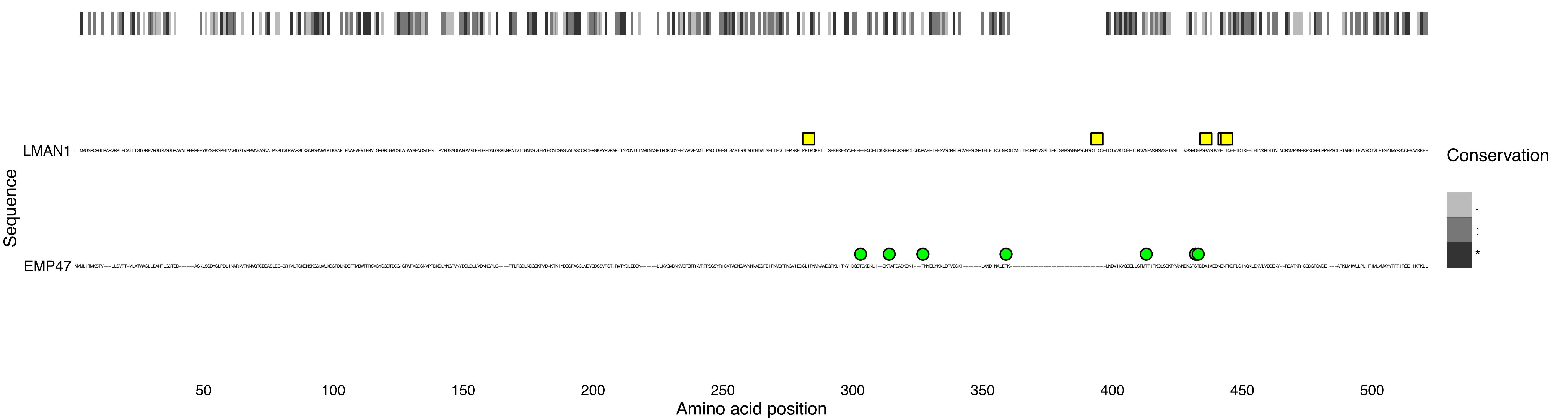
LMAN1
EMP47
Sequence
Conservation
.
:
*
50
100
150
200
250
300
350
400
450
500
Amino acid position

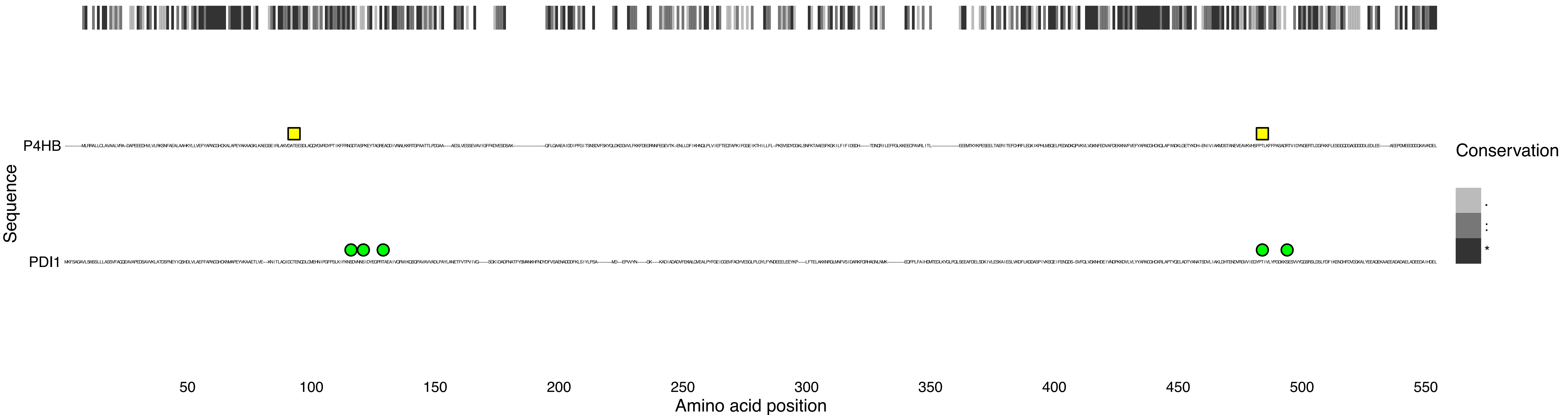
P4HB
PDI1
Sequence
Conservation
.
:
*
50
100
150
200
250
300
350
400
450
500
550
Amino acid position

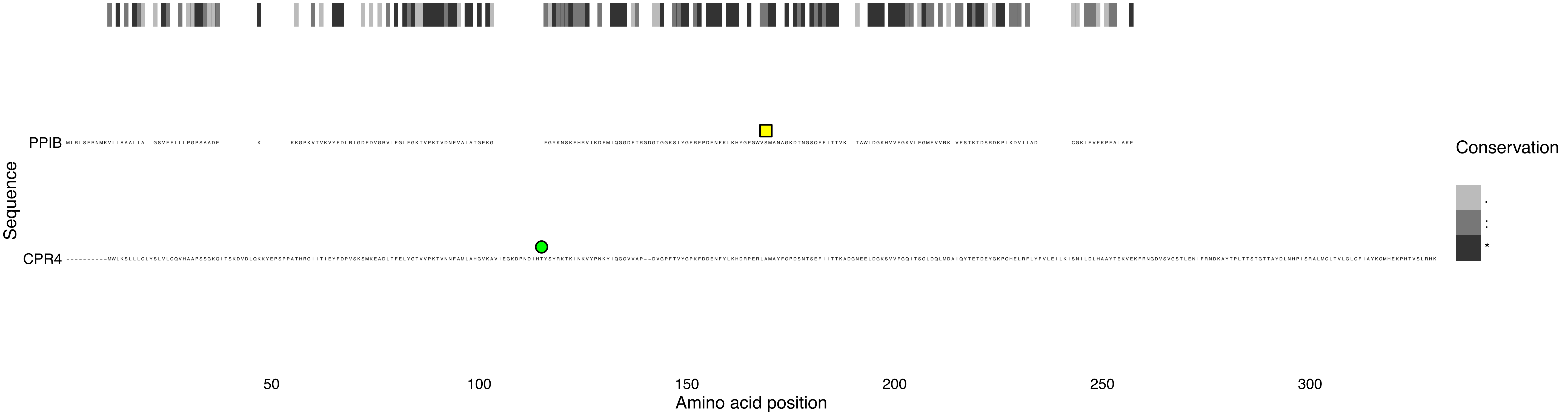

PPIB
CPR4
Sequence
Conservation
.
:
*
50
100
150
200
250
300
Amino acid position

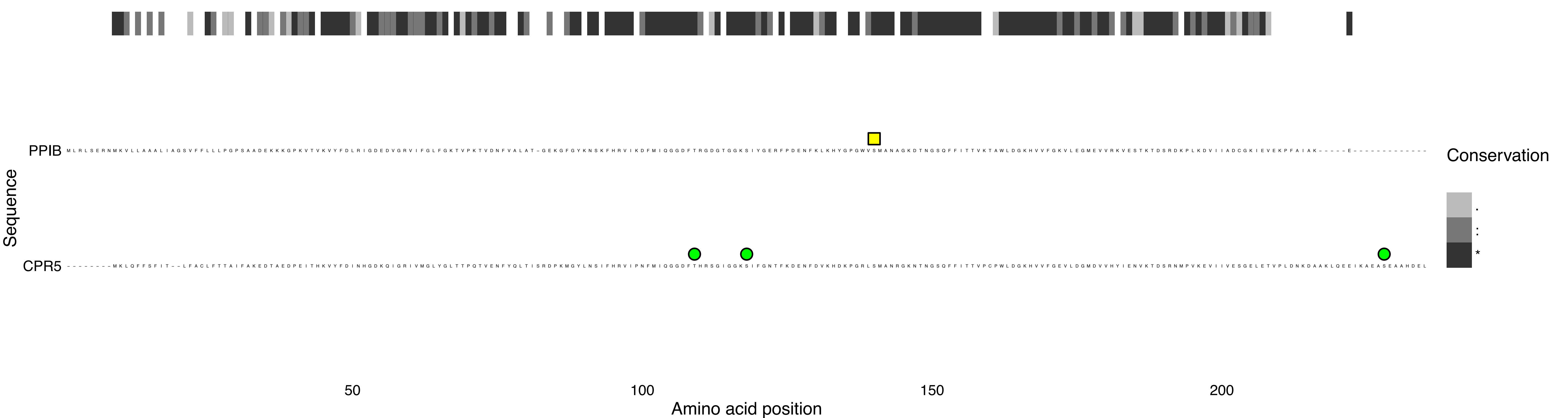
PPIB
CPR5
Sequence
Conservation
.
:
*
50
100
150
200
Amino acid position

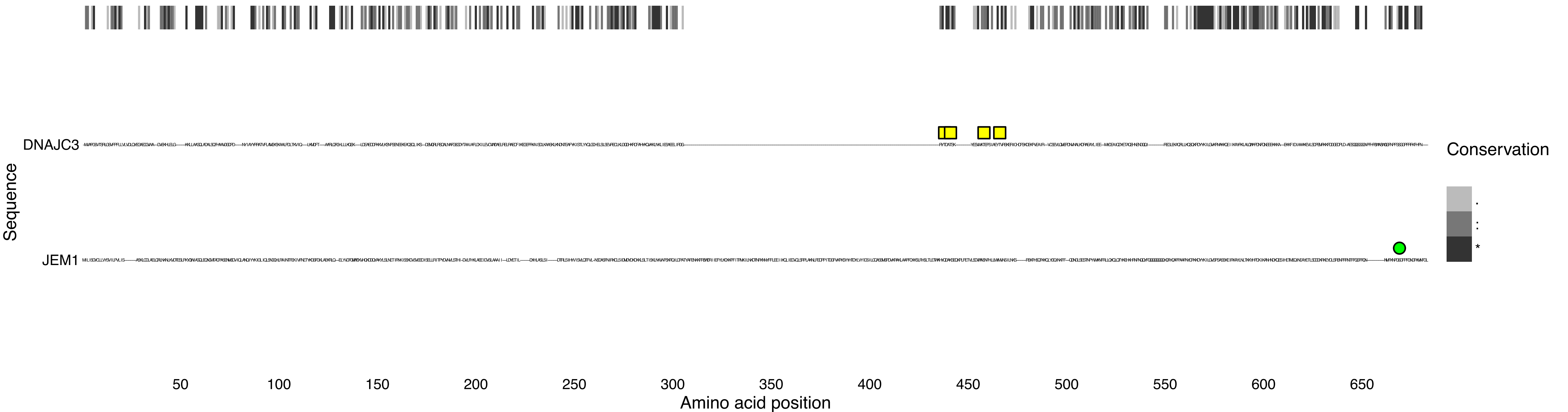
DNAJC3
JEM1
Sequence
Conservation
.
:
*
50
100
150
200
250
300
350
400
450
500
550
600
650
Amino acid position

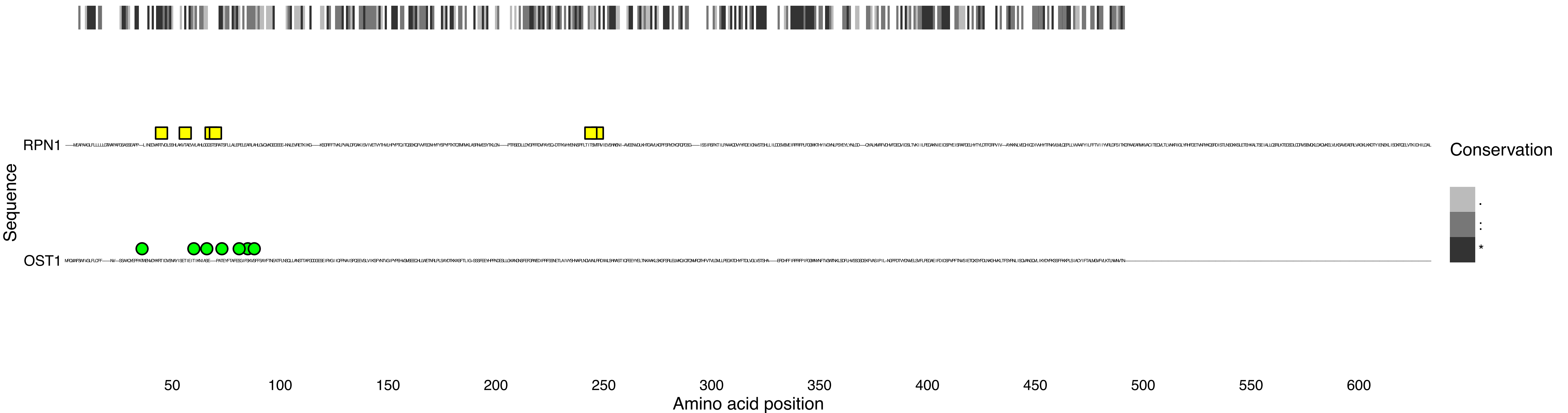

RPN1
OST1
Sequence
Conservation
.
:
*
50
100
150
200
250
300
350
400
450
500
550
600
Amino acid position

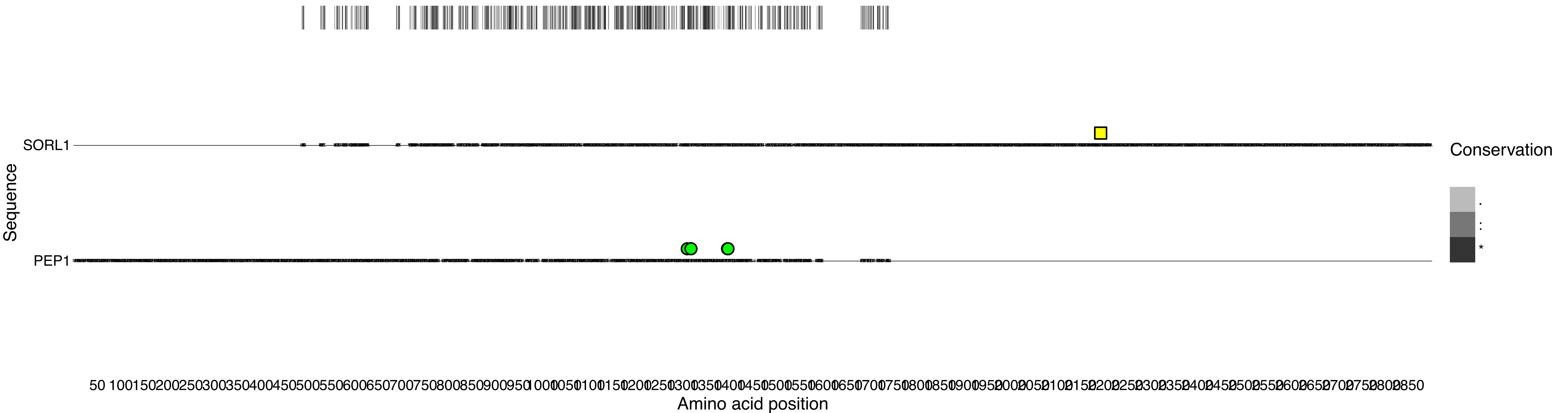
SORL1
PEP1
Sequence
Conservation
.
:
*
Amino acid position

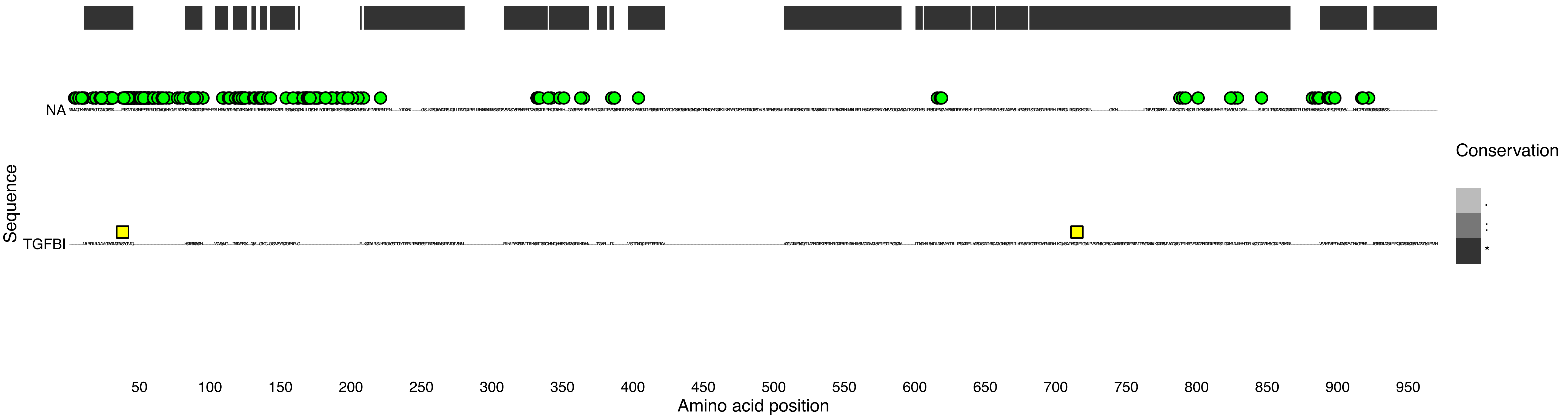
NA
TGFBI
Sequence
Conservation
.
:
*
50
100
150
200
250
300
350
400
450
500
550
600
650
700
750
800
850
900
950
Amino acid position

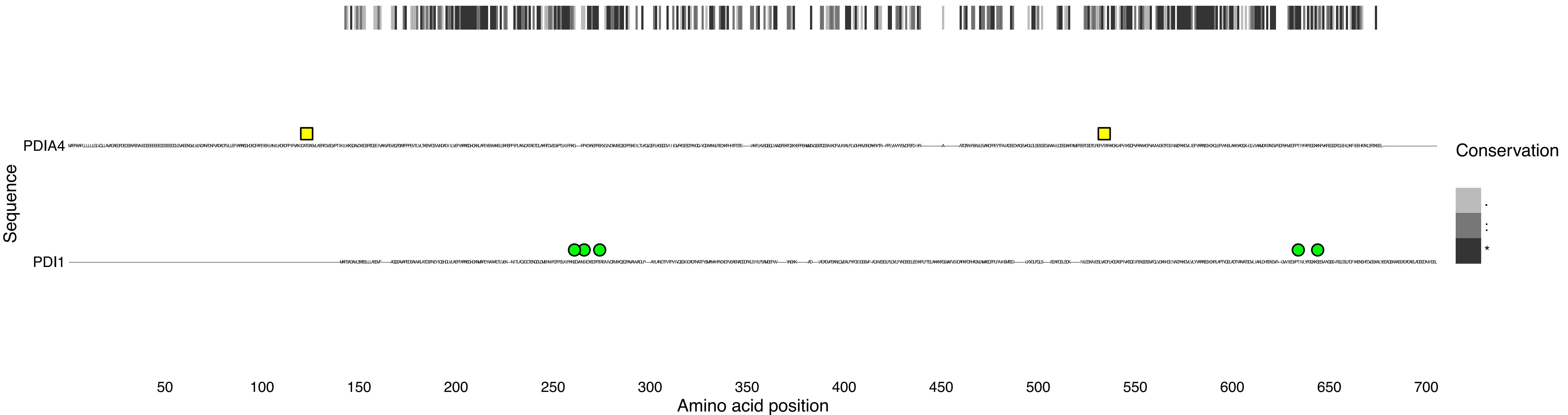

PDIA4
PDI1
Sequence
Conservation
.
:
*
50
100
150
200
250
300
350
400
450
500
550
600
650
700
Amino acid position

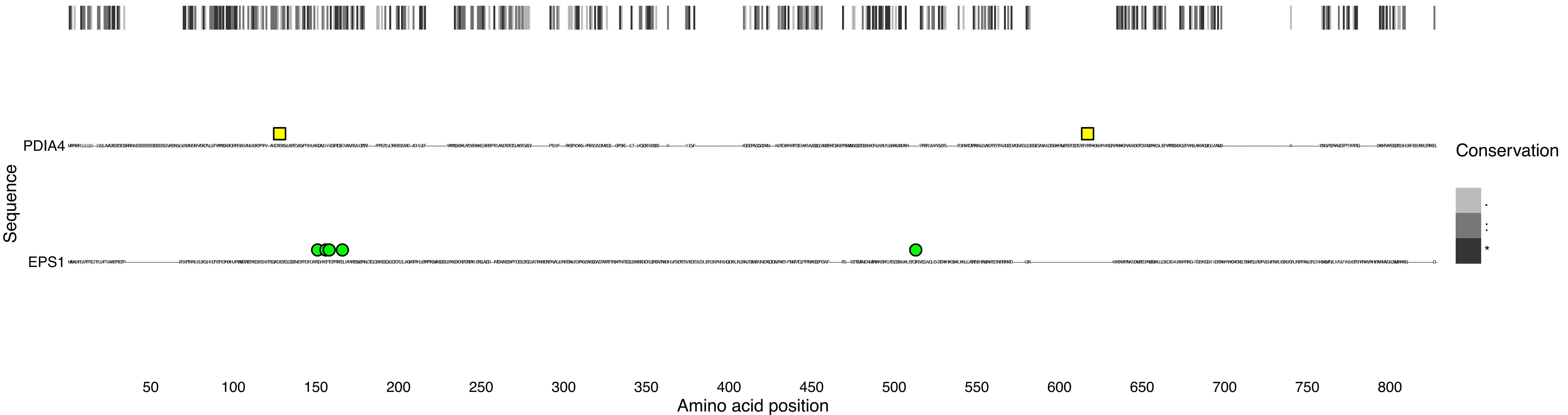
PDIA4
EPS1
Sequence
Conservation
.
:
*
50
100
150
200
250
300
350
400
450
500
550
600
650
700
750
800
Amino acid position

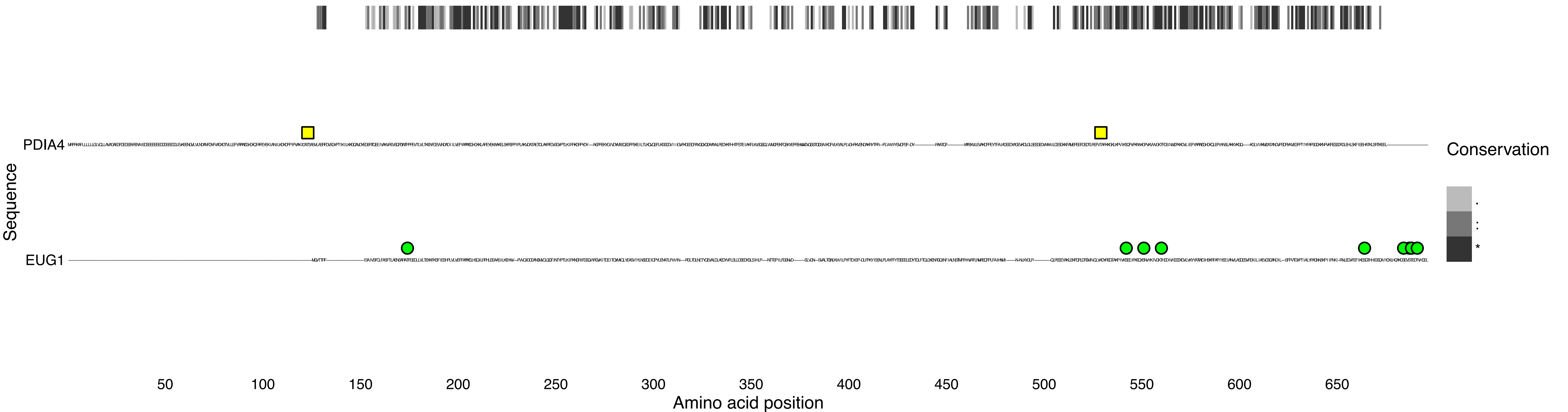
PDIA4
EUG1
Sequence
Conservation
.
:
*
50
100
150
200
250
300
350
400
450
500
550
600
650
Amino acid position

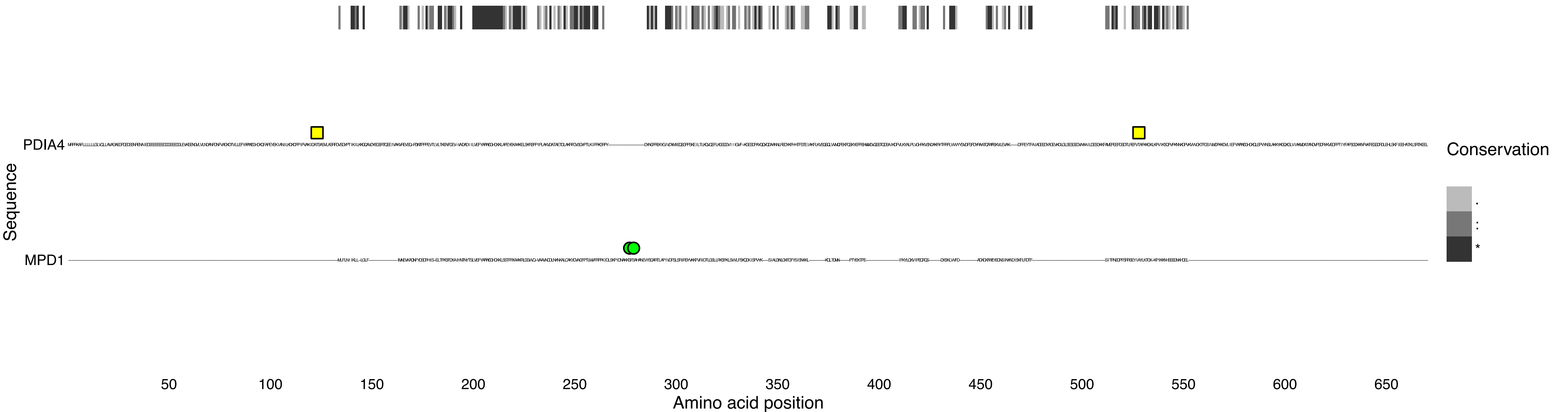
PDIA4
MPD1
Sequence
Conservation
.
:
*
50
100
150
200
250
300
350
400
450
500
550
600
650
Amino acid position

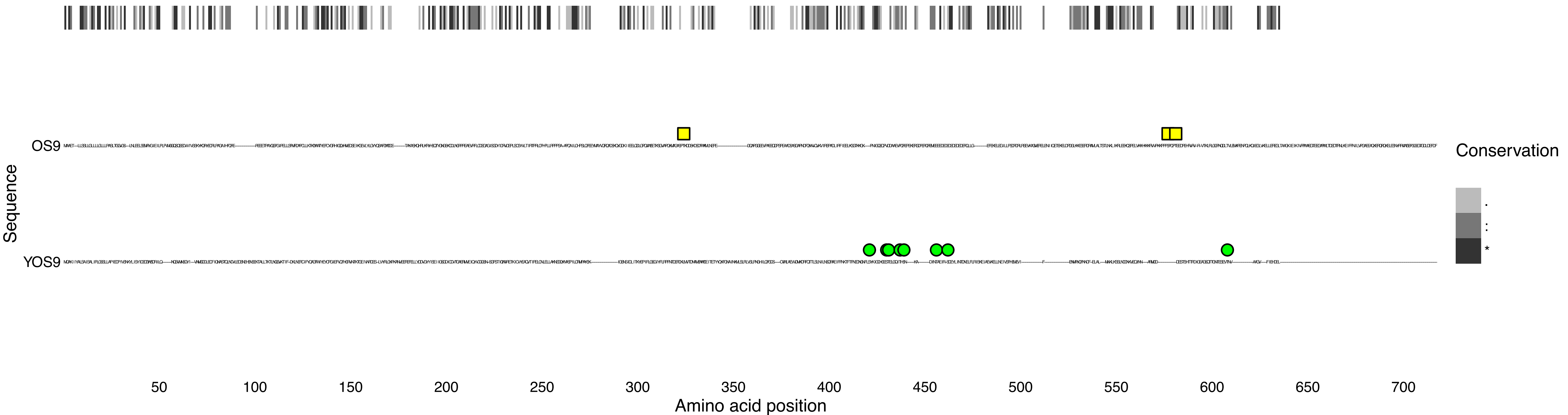

OS9
YOS9
Sequence
Conservation
.
:
*
50
100
150
200
250
300
350
400
450
500
550
600
650
700
Amino acid position

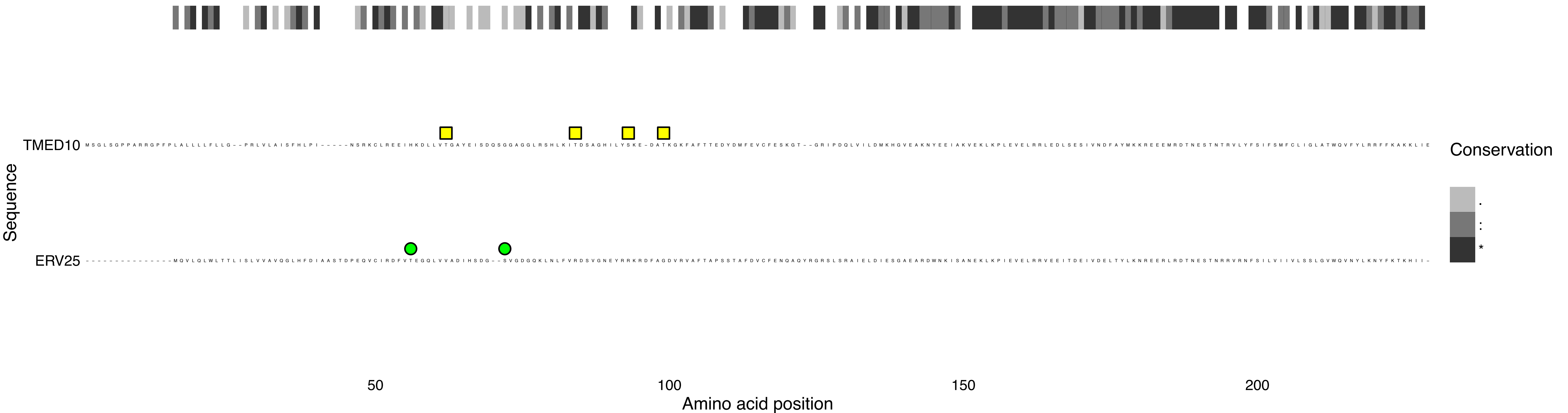

TMED10
ERV25
Sequence
Conservation
.
:
*
50
100
150
200
Amino acid position

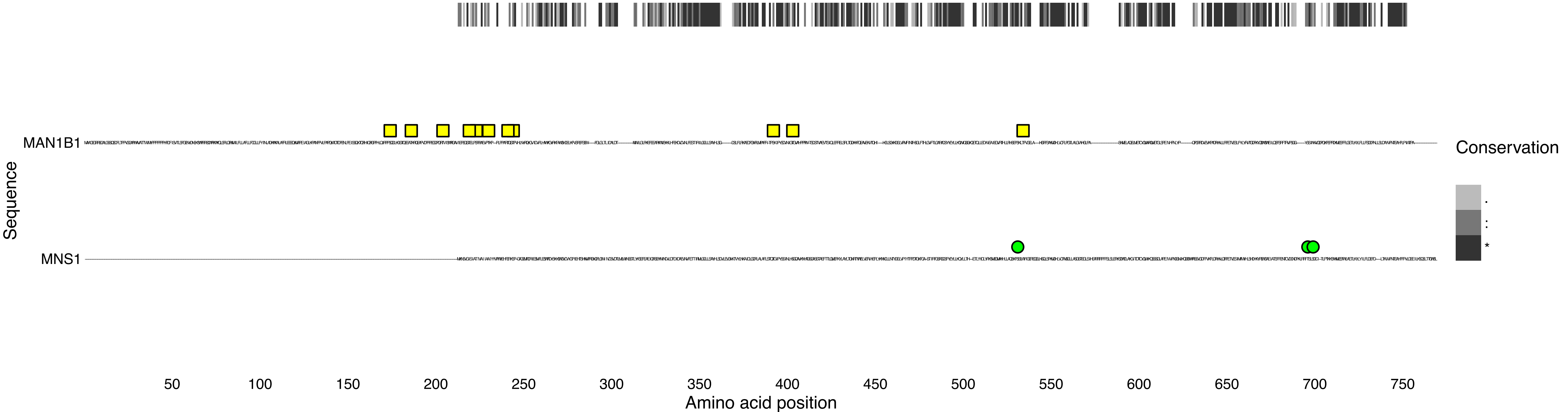
MAN1B1
MNS1
Sequence
Conservation
.
:
*
50
100
150
200
250
300
350
400
450
500
550
600
650
700
750
Amino acid position

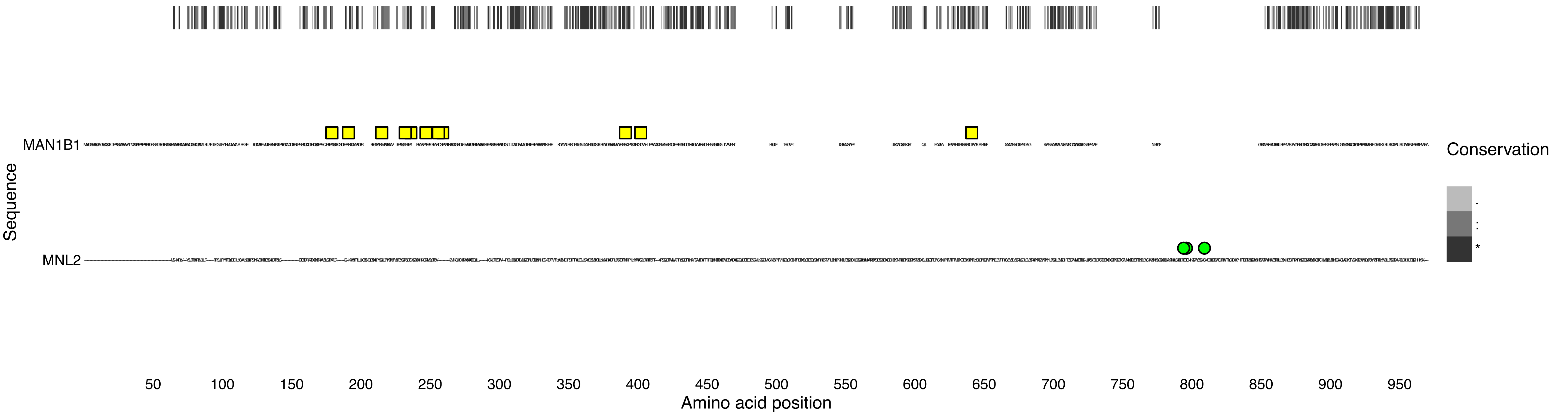

MAN1B1
MNL2
Sequence
Conservation
.
:
*
50
100
150
200
250
300
350
400
450
500
550
600
650
700
750
800
850
900
950
Amino acid position

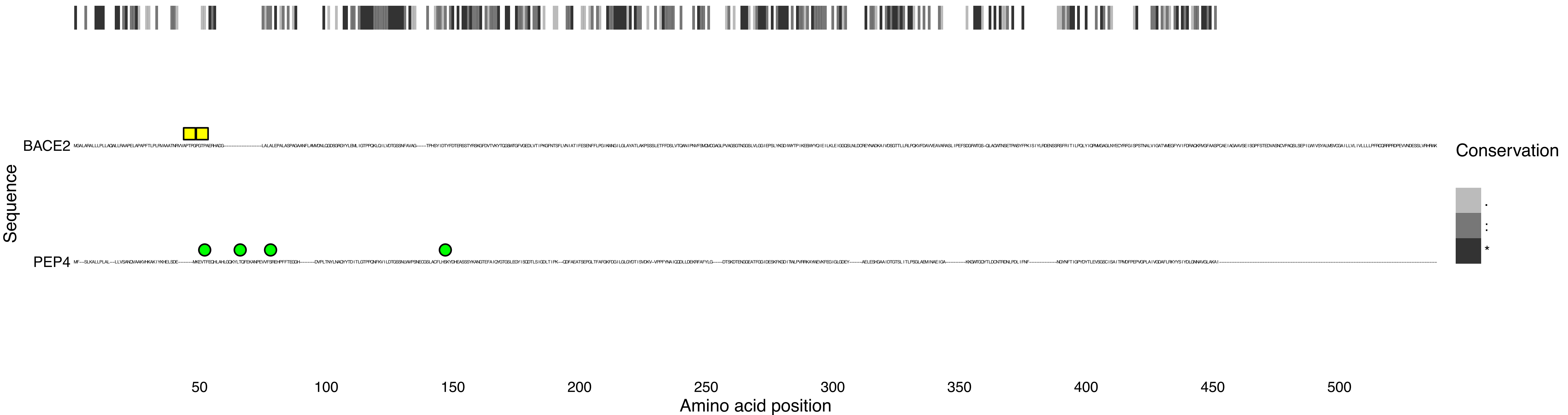

BACE2
PEP4
Sequence
Conservation
.
:
*
50
100
150
200
250
300
350
400
450
500
Amino acid position

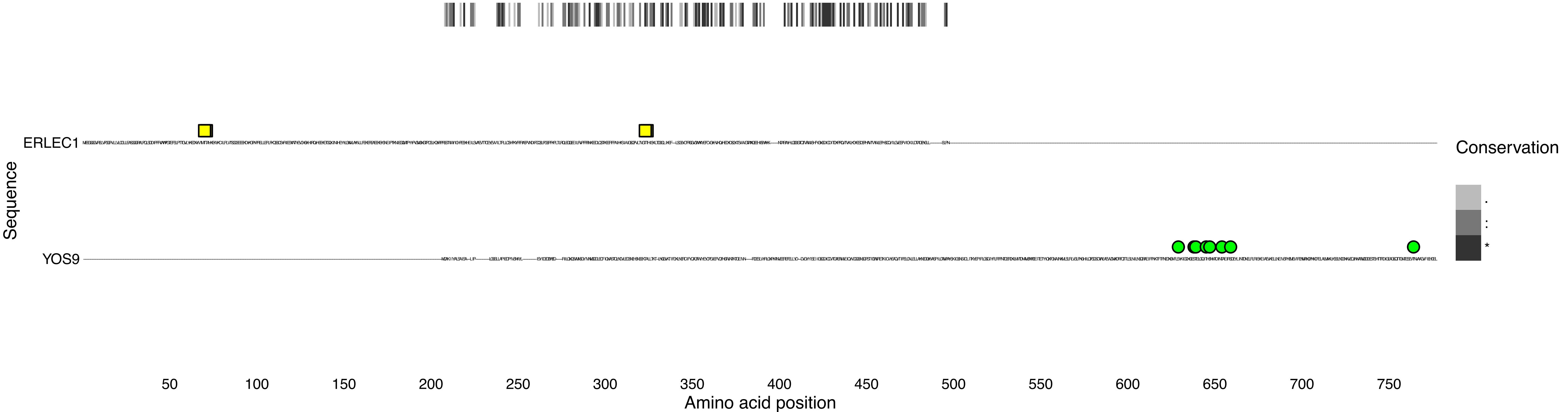
ERLEC1
YOS9
Sequence
Conservation
.
:
*
50
100
150
200
250
300
350
400
450
500
550
600
650
700
750
Amino acid position

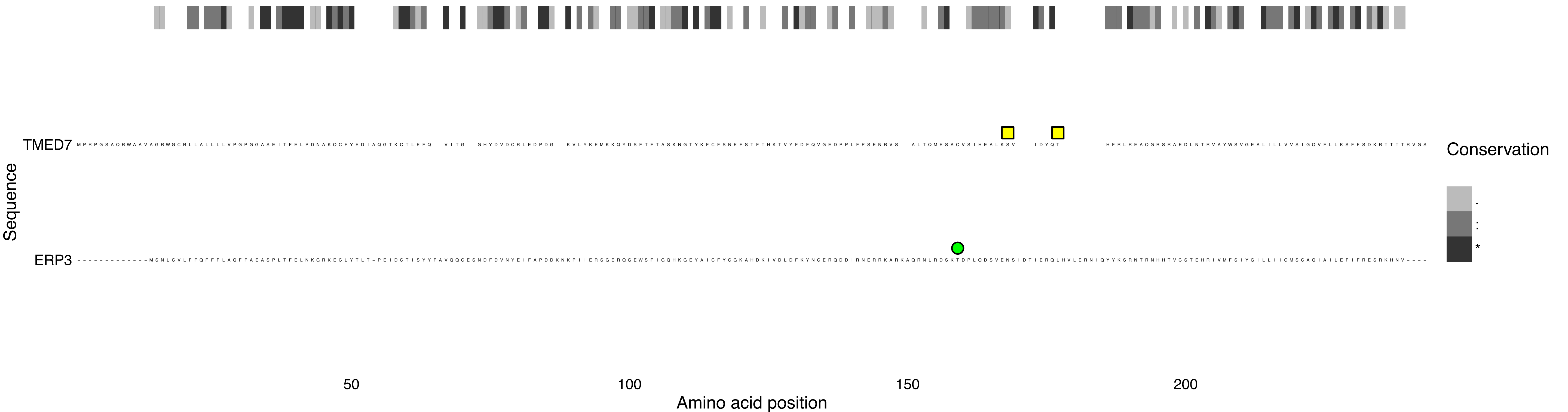
TMED7
ERP3
Sequence
Conservation
.
:
*
50
100
150
200
Amino acid position

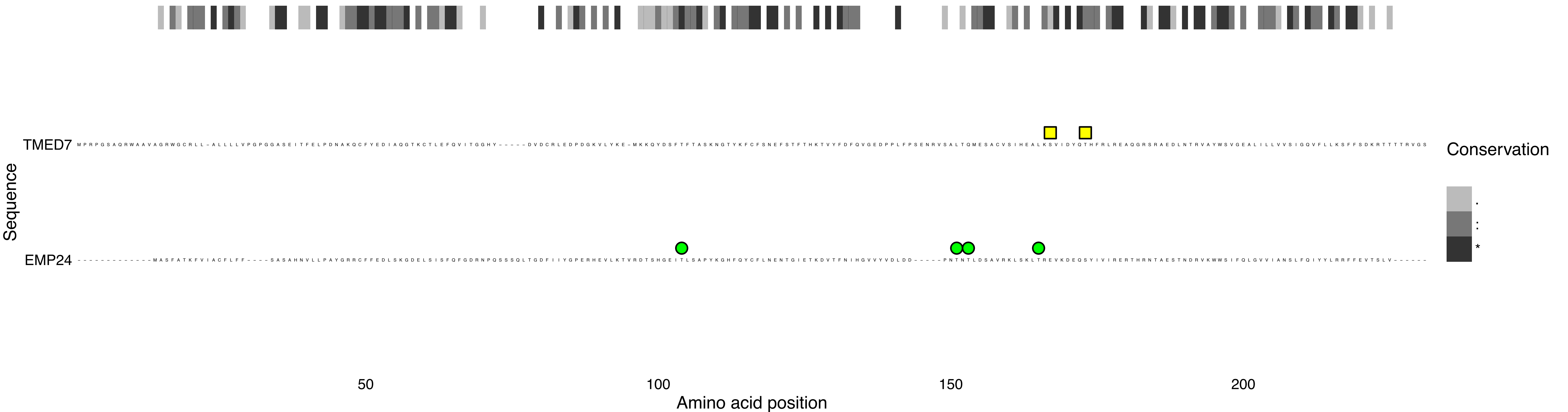
TMED7
EMP24
Sequence
Conservation
.
:
*
50
100
150
200
Amino acid position

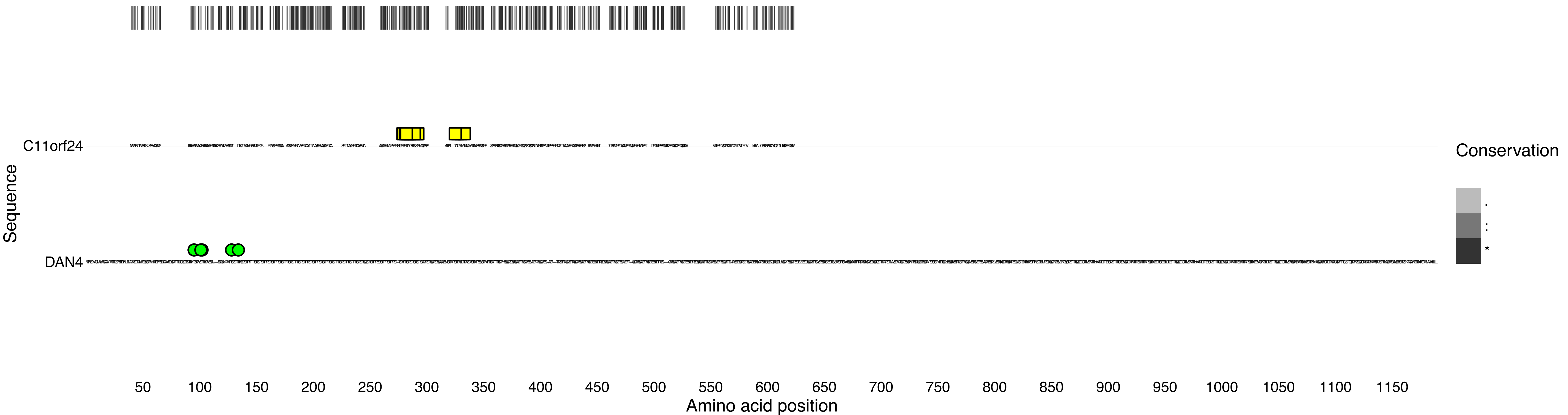
C11orf24
DAN4
Sequence
Conservation
.
:
*
50
100
150
200
250
300
350
400
450
500
550
600
650
700
750
800
850
900
950
1000
1050
1100
1150
Amino acid position

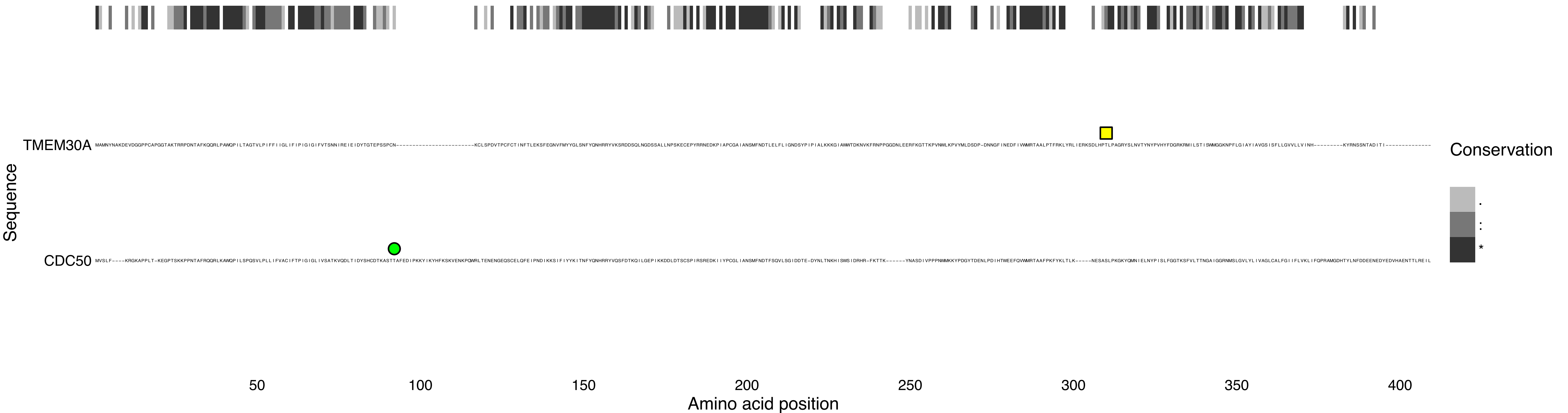
TMEM30A
CDC50
Sequence
Conservation
.
:
*
50
100
150
200
250
300
350
400
Amino acid position

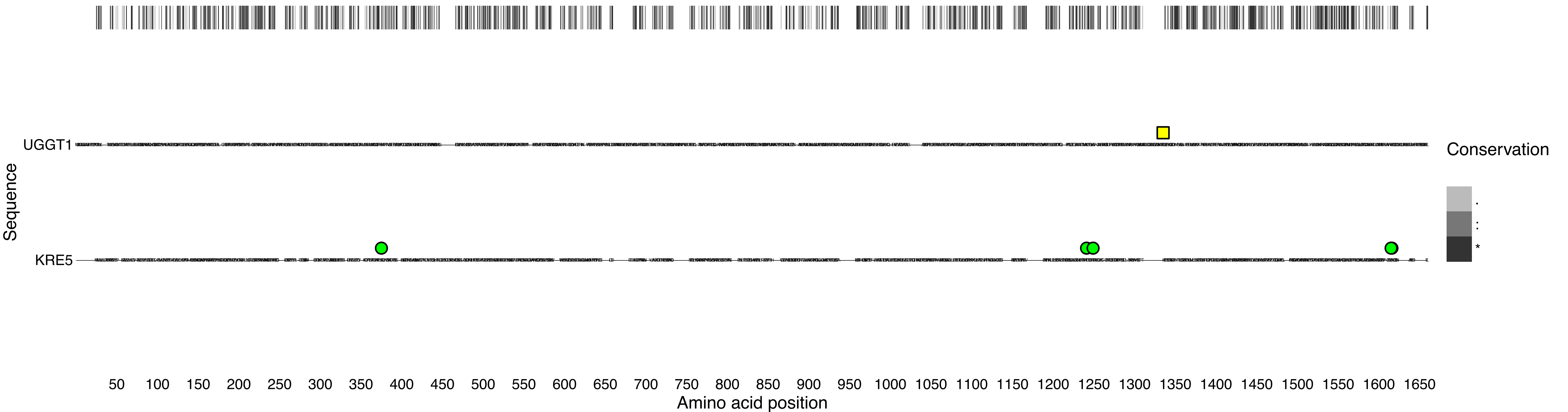

UGGT1
KRE5
Sequence
Conservation
.
:
*
50
100
150
200
250
300
350
400
450
500
550
600
650
700
750
800
850
900
950
1000
1050
1100
1150
1200
1250
1300
1350
1400
1450
1500
1550
1600
1650
Amino acid position

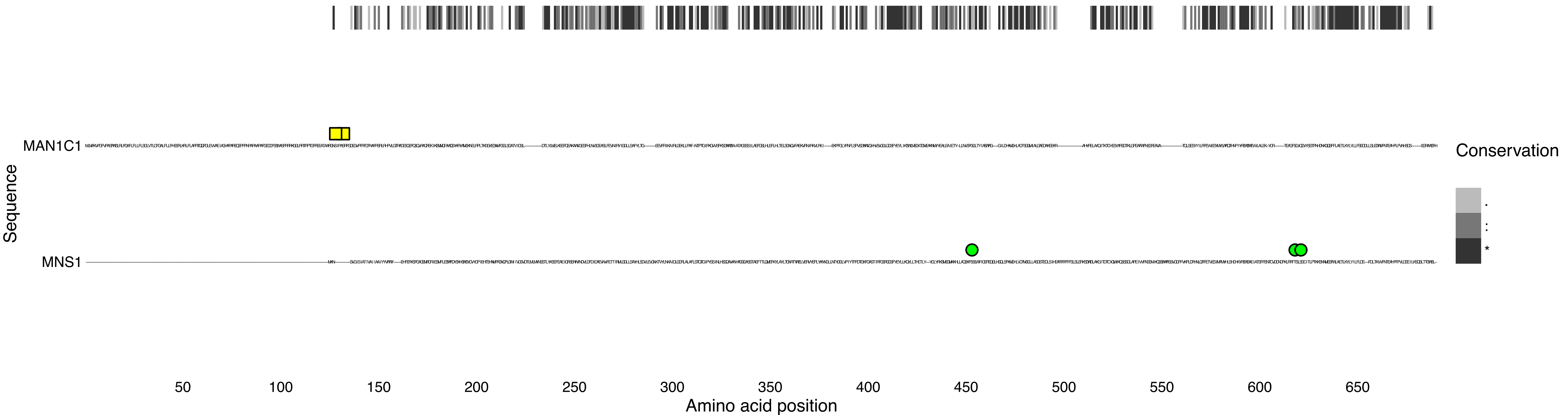
MAN1C1
MNS1
Sequence
Conservation
.
:
*
50
100
150
200
250
300
350
400
450
500
550
600
650
Amino acid position

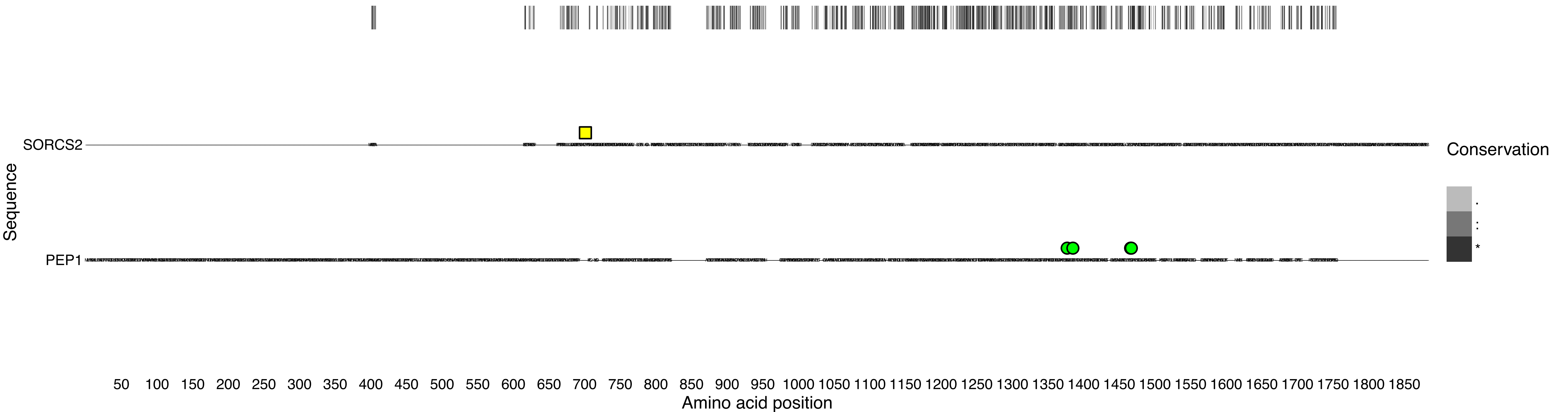

SORCS2
PEP1
Sequence
Conservation
.
:
*
50 100 150 200 250 300 350 400 450 500 550 600 650 700 750 800 850 900 950 1000 1050 1100 1150 1200 1250 1300 1350 1400 1450 1500 1550 1600 1650 1700 1750 1800 1850
Amino acid position

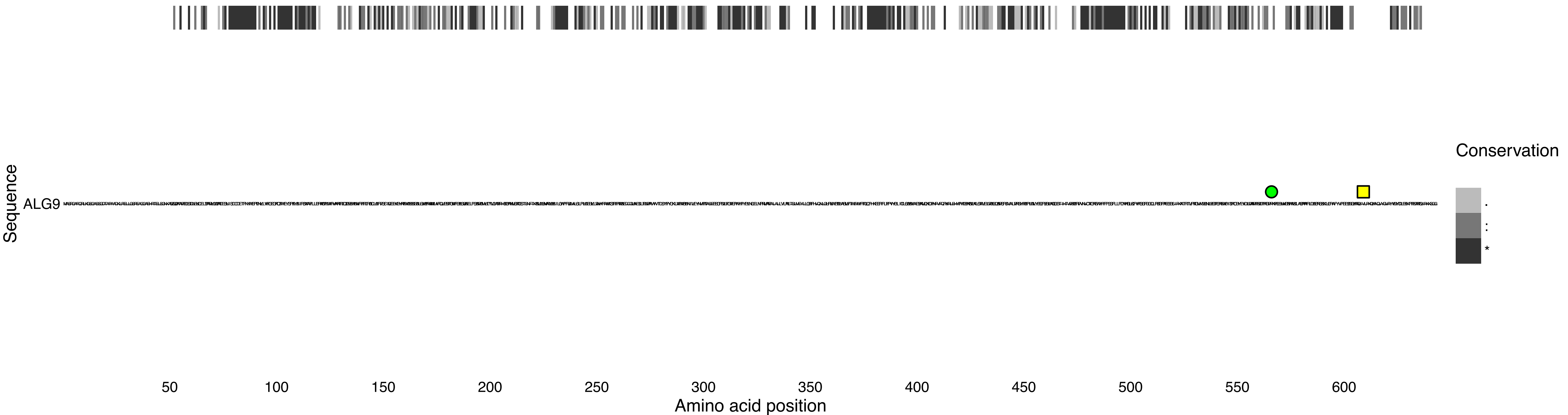
Conservation
.
:
*
Sequence
ALG9
50
100
150
200
250
300
350
400
450
500
550
600
Amino acid position

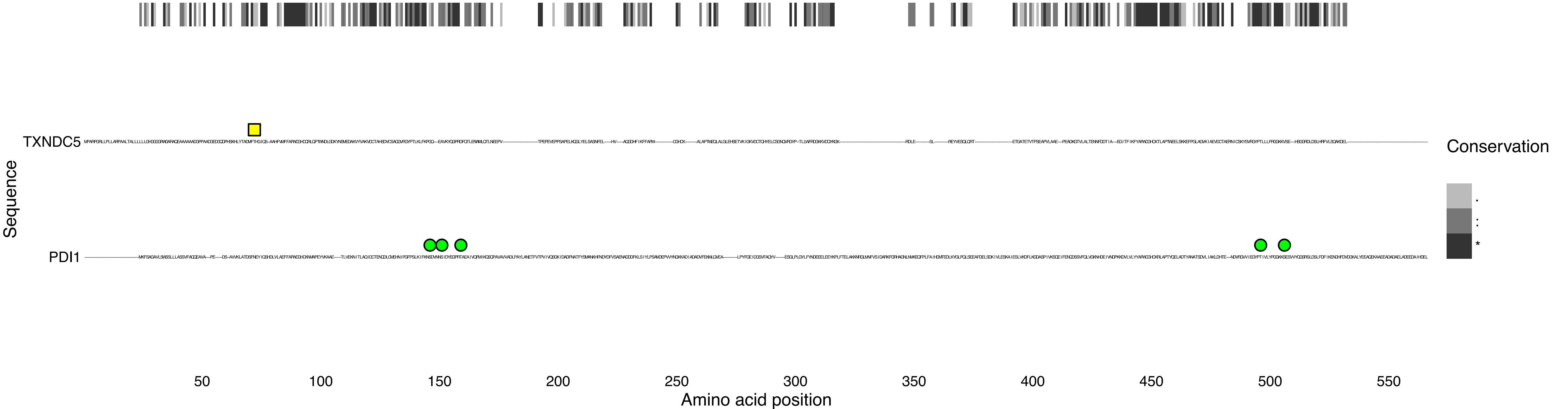

TXNDC5
PDI1
Sequence
Conservation
.
:
*
50
100
150
200
250
300
350
400
450
500
550
Amino acid position

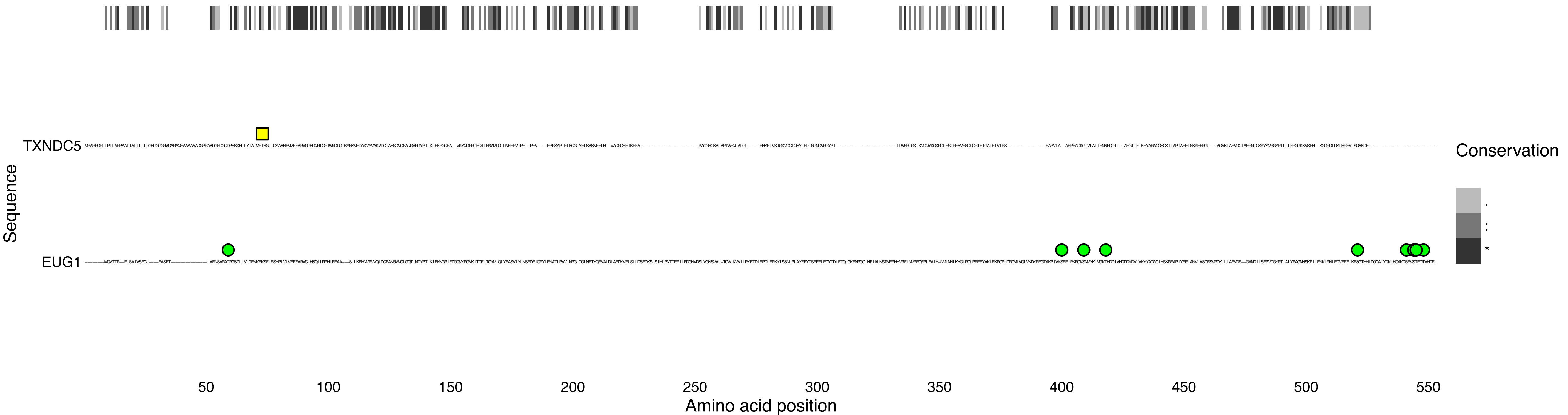

TXNDC5
EUG1
Sequence
Conservation
.
:
*
50
100
150
200
250
300
350
400
450
500
550
Amino acid position

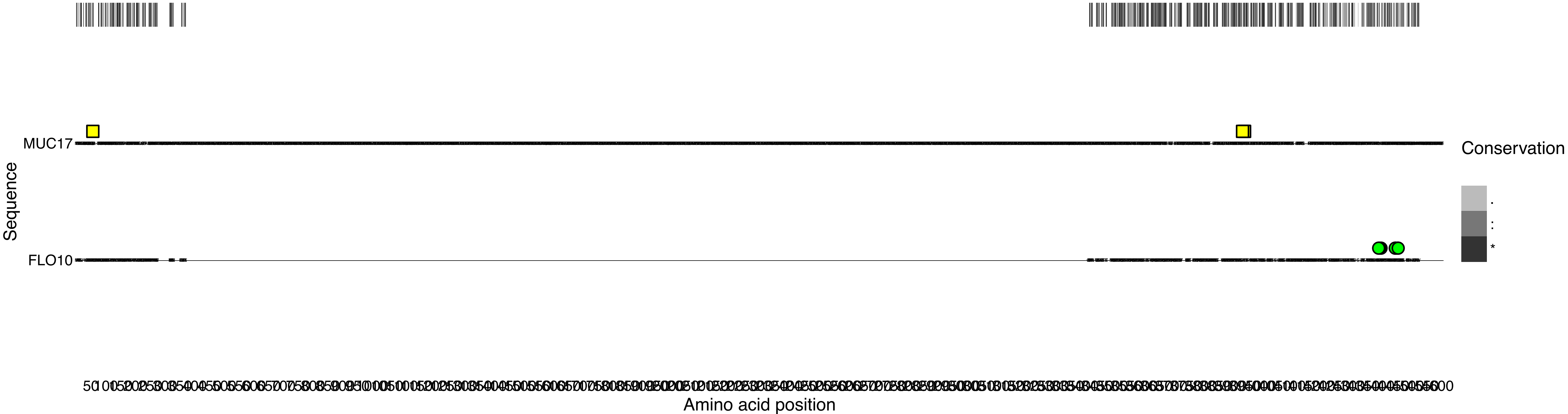
MUC17
FLO10
Sequence
Conservation
.
:
*
Amino acid position

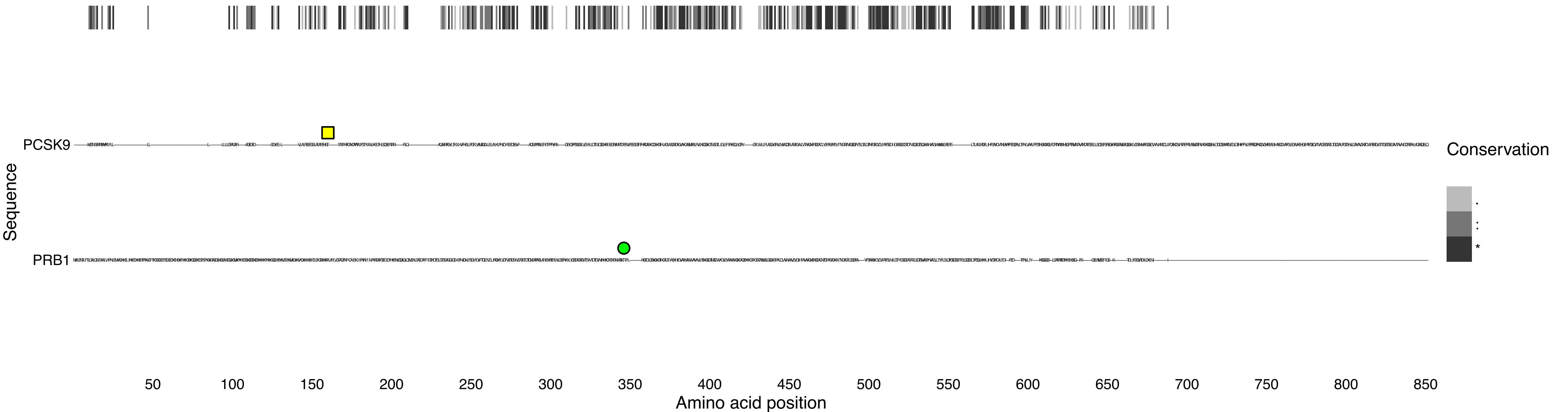
PCSK9
PRB1
Sequence
Conservation
.
:
*
50
100
150
200
250
300
350
400
450
500
550
600
650
700
750
800
850
Amino acid position
